# Immunogenomic pathways associated with cytotoxic lymphocyte infiltration and survival in colorectal cancer

**DOI:** 10.1186/s12885-020-6513-4

**Published:** 2020-02-14

**Authors:** Yuanyuan Shen, Yue Guan, Justin J. Hummel, Chi-Ren Shyu, Jonathan B. Mitchem

**Affiliations:** 1 Harry S. Truman Memorial Veterans’ Hospital, Columbia, MO USA; 2 Institute for Data Science and Informatics, University of Missouri, 238 Naka Hall, Columbia, MO 65211-2060 USA; 3 Department of Surgery, University of Missouri School of Medicine, 1 Hospital Dr., Columbia, MO 65212 USA

**Keywords:** Colorectal cancer, Colon, Rectum, Immunotherapy, Immunogenomics, Immuno-oncology, Cytotoxic lymphocytes

## Abstract

**Background:**

Colorectal cancer (CRC) is the second leading cancer killer in the US today and patients with metastatic disease have only a 14% 5-year survival. One of the most impactful recent advances in cancer therapy, immune checkpoint inhibition, has not been shown to be effective for the majority of these patients. In this study, we use The Cancer Genome Atlas (TCGA) and recently developed informatic-based tools to identify targets for immune based therapy in colorectal cancer patients.

**Methods:**

Open access, pre-processed (level 3) mRNA data and clinical data from colorectal patients from the TCGA was downloaded from FireCloud. Using the Microenvironment Cell Populations-Counter method (MCP-Counter), cytotoxic lymphocyte scores were calculated for all patients. Patients were then grouped by cytotoxic lymphocyte score (High vs Low), pathologic stage, and location to identify differentially expressed genes. Pathway enrichment analysis was performed using Reactome to determine differentially expressed genes associated with immune pathways. Survival analysis was performed with identified differentially expressed genes.

**Results:**

In the TCGA dataset, there are 461 colon and 172 rectal cancer patients. After stratifying patients by cytotoxic lymphocyte score, anatomical location, and stage, we found a significant number of differentially expressed genes. We identified one pathway, “immunoregulatory interactions between a lymphoid and non-lymphoid cell”, that was highly enriched and included in all tumor locations and stages. Survival analysis performed with differentially expressed genes in this pathway identified 21 different genes associated with survival and cytotoxic lymphocyte infiltration, with ~ 70% of these genes occurring in the metastatic right-sided CRC group. Specifically, all genes associated with survival in the metastatic right-sided colorectal cancer group with low cytotoxic lymphocyte scores positively impacted survival.

**Conclusions:**

Utilizing the TCGA, a publicly available dataset, and informatics-based analyses, we identified potential targets to improve immune based therapy in colorectal cancer. Additionally, we note the most targets in metastatic right-sided CRC patients, the patient group with the worst predicted survival. The results from this study demonstrate the ability of informatics-based analytic techniques to identify new therapeutic targets as well as improve patient selection for intervention, helping us to achieve the goals of precision-based oncology.

## Background

Despite recent advances in detection and therapy, colorectal cancer (CRC) remains the second leading cause of cancer-related death in the US [1]. Immune based therapies such as immune checkpoint inhibition have recently made significant advances in a number of difficult to treat malignancies like non-small cell lung cancer, melanoma, and renal cell cancer [2]. However, these results have not yet extended to the majority of patients with CRC [3]. This is despite significant data that anti-tumor immunity is important for prognosis and treatment response in these patients [4, 5]. This suggests that there is significant progress to be made in the application of immune based therapy in CRC.

When considering immune based treatments, a critical factor is effective tumor infiltration of cytotoxic lymphocytes [6]. In colorectal cancer, this is evident as patients who demonstrate response to immune checkpoint inhibition and currently have an FDA approved indication for this therapy, are those with microsatellite instability-high (MSI-H) tumors [7]. These tumors are characterized by high mutational load, neoepitope formation, and an intense lymphocytic infiltrate when compared to microsatellite stable (MSS) tumors [8]. Microsatellite instability-high tumors, however, are also associated with increased mutations in immune related genes and expression of negative regulatory genes, demonstrating that tumors try to dampen the immune response by multiple pathways [9, 10]. Additionally, recent studies have suggested that the use of other markers including lymphocyte infiltration and tumor mutational burden may better predict survival and the potential for response to immune based therapy [5, 11]. It is therefore critical to develop a better understanding of immune resistance mechanisms to improve therapy in colorectal cancer patients.

In the current era of precision medicine, research is concentrated on providing more effective treatments by focusing on patient specific factors. This is particularly important in colorectal cancer, as subsets of patients responsive to targeted therapy, immune-based therapy, and chemotherapy have previously been identified [3, 12, 13]. Colorectal cancer, however, is a heterogeneous disease made up of multiple subgroups [14]. Even simple clinical characteristics often overlooked in molecular studies, such as anatomic location, are important for prognosis [15, 16]. Despite these differences in subtype and clinical characteristics, T lymphocyte infiltration has been demonstrated to be important for prognosis [5].

Data repositories such as The Cancer Genome Atlas (TCGA) allow for the in-depth study of patients on a molecular and clinical basis [17]. Recently, a novel computational method for predicting the abundance of different cells within the tumor microenvironment using RNA-seq data was developed and validated with histologic specimens called the Microenvironment Cell Population–Counter (MCP-Counter, [4]). This method allows for an effective comparison of the composition and pathways associated with cellular infiltration in the tumor microenvironment, improving over other methods primarily based on microarray data and gene set enrichment analysis. In this study, we use the MCP-counter program to create tumor cytotoxic lymphocyte (CL) abundance scores. After grouping patients based on cytotoxic lymphocyte abundance score, stage, and tumor location, we found one immune pathway that was highly enriched at all tumor locations and stages, the “Immunoregulatory interactions between a Lymphoid and a non-Lymphoid cell” pathway, suggesting specific targets to improve immune based therapy in colorectal cancer patients.

## Methods

### The cancer genome atlas data access and processing

**Fig. 1:**
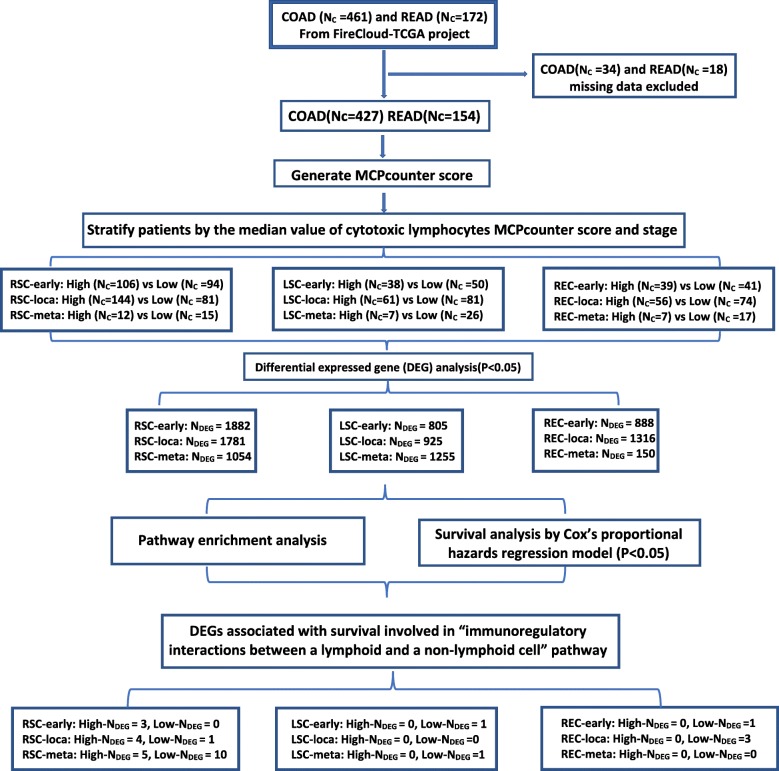
An outline of the methods and organization of this study. This flowchart describes the process and breakdown of patients into anatomical location groups and pathological stages based on cytotoxic lymphocytes (CL) abundance score. After dividing patients into anatomical groups and stages, we then compared CL-High and CL-Low to determine differential expressed genes (DEGs). Pathway enrichment and Survival analysis was then undertaken using identified DEGs. Pathway associations were determined for DEGs using the Reactome online browser. CL, cytotoxic lymphocytes; COAD, colon adenocarcinoma; READ, rectal adenocarcinoma; DEG, Differentially Expressed Gene; Nc, the number of cases; Ng, the number of genes; RSC, right-sided colon cancer; LSC, left-sided colon cancer; REC, rectal cancer

The data shown here is based upon data originally generated and organized by FireCloud from the Broad Institute (Fig. 1). Full permission access transcriptomic data was obtained from dbGAP. We downloaded CRC patients’ open access, pre-processed mRNA expression data (level 3 data, [18]) from both platforms, Illumina-HiSeq and Illumina-GA; as well as mRNA RNA-Seq by Expectation Maximization (RSEM) normalized data; and patients’ clinical data from the cohorts TCGA_COAD_ControlledAccess and TCGA_READ_ControlledAccess by gsutil Tool.

We integrated pertinent clinical data (age, gender, microsatellite status, anatomical location, pathologic stage, Tumor Node and Metastasis (TNM) classification, days to last follow up, and vital status), and RNA-seq by participant ID. Each CRC patient has pre-identified microsatellite status labeled as “microsatellite instability test results”. Thirty-four patients with colon cancer and 18 patients with rectal cancer were excluded due to missing information including indeterminate microsatellite status, unclear anatomical location or pathologic stage, and unmatched RNA-seq data. Then for every patient, we implemented the R package Microenvironment Cell Populations-Counter on the RNA-Seq by Expectation Maximization (RSEM) normalized RNA-seq data to create cell type abundance scores [4]. There are 10 cell populations simultaneously quantified in the tumor microenvironment, including 8 immune cell populations (T cells, CD8 T cells, Cytotoxic lymphocytes, NK cells, B lineage, Monocytic lineage, Myeloid dendritic cells, Neutrophils), endothelial cells and fibroblasts. Specifically, the gene set for cytotoxic lymphocytes includes the genes: CD8A, EOMES, FGFBP2, GNLY, KLRC3, KLRC4, and KLRD1. We used the median value of the cytotoxic lymphocytes (CL) score to group the patients into high (≥ median value, CL-High) and low (< median value, CL-Low) groups. We then grouped patients by anatomical location and stratified by cytotoxic lymphocyte score and pathologic stage. For consistency in clinical intervention while increasing group size, we assigned pathologic stage I-II to “early” stage, stage I-III as “localized”, and stage IV as “metastatic” (Fig. 1). Patients were grouped by tumor subsite: tumors located in the cecum, ascending colon, hepatic flexure, and transverse colon were categorized as having right-sided colon cancer (Fig. 1, RSC); tumors located in the splenic flexure, descending colon, sigmoid colon or rectosigmoid junction were categorized as having left-sided colon cancers (Fig. 1, LSC); patients with tumors located in the rectum were kept in this group (Fig. 1, REC).

### Demographics and clinicopathologic characteristic analysis

Demographic, clinical, and pathologic characteristics were retrieved as stated above. Statistical analyses were performed using Prism 7 (GraphPad Software). Patients’ basic clinical features were summarized by descriptive statistics, including means and standard deviation, and an unpaired t-test was used for normally distributed continuous data. Categorical variables were compared using Fisher’s exact and chi-square tests. A *p* value < 0.05 was considered statistically significant.

### RNA-seq differential gene expression analysis

RNA-seq differential gene expression analysis was performed with the edgeR package using the raw data downloaded from the Illumina- HiSeq and Illumina-GA platforms [19]. Differentially expressed genes were defined as genes with an absolute fold change > 1 between patients with high and low cytotoxic lymphocyte scores with a *p* value < 0.05 [19]. Genes with Benjamini-Hochberg adjusted False Discovery Rate (FDR) < 0.05 were considered to be significantly differentially expressed for further steps. For each cohort, we identified 20,531 total genes by RNA-seq raw counts. The Reactome online browser was used to identify immune functional differentially expressed genes [20]. Figure 1 outlines this process.

### Pathway enrichment and survival analysis

Pathway enrichment analysis was performed to evaluate the pathways associated with differentially expressed genes. The genes included in the MCP-counter cytotoxic lymphocyte gene panel (CD8A, EOMES, FGFBP2, GNLY, KLRC3, KLRC4, and KLRD1) were excluded from pathway enrichment analysis. Dotplot was used to illustrate the comparison of enriched Reactome pathways among differentially expressed genes in each location and stage. These results were analyzed by clusterProfiler, DOSE, and ReactomePA R packages. Next, we performed survival analyses using the identified differentially expressed genes. Patients were organized by stage and location as outlined above. The normalized RNA-seq data of differentially expressed genes used for survival analysis was processed using the Survival R package [20]. For each differentially expressed gene, if the normalized gene expression value was more than the median level, we labeled it as “high,” and otherwise as “low.” The Kaplan–Meier survival curves generated were assessed by the Cox regression model for each immune functional differentially expressed gene using the Survminer R package [21, 22]. The survival curves of patients with high gene expression and low gene expression were compared by log-rank test. For each patient, overall survival (OS) was used as the endpoint, either the days from diagnosis to death, or to the last follow-up (Fig. 1).

The Reactome pathway online browser was used to identify differentially expressed genes associated with survival in sub-pathways of “immunoregulatory interactions between a lymphoid and a non-lymphoid cell pathway” (Fig. 1).

## Results

### Patient characteristics

In the most recently updated TCGA dataset (June 01, 2016), there are 461 colon cancer (COAD) and 172 rectal cancer (READ) cases (Fig. 1, [17]). Thirty-four patients with colon cancer and 18 patients with rectal cancer were excluded due to missing information. The RNA-seq data with matched clinical data were integrated from COAD patients (Nc = 427) and READ patients (Nc = 154). Cytotoxic lymphocyte abundance scores were generated using the MCP-counter method [4]. Patients were then separated based on the median cytotoxic lymphocyte score (26.72; 95% CI: 24.1–30.1, Additional file 1: Table S1, values and analysis included in Additional file 2). Patients with cytotoxic lymphocyte scores ≥ the median were classified as cytotoxic lymphocyte-high (CL-High) and those with scores < the median were classified as cytotoxic lymphocyte-low (CL-Low). We then confirmed that there was a significant difference in cytotoxic lymphocyte scores between CL-High and CL-Low groups (73.66 ± 64.2 v 14.07 ± 6.69, *p* < 0.0001, Additional file 1: Figure S1). Colorectal cancer patients were then separated by anatomical location, cytotoxic lymphocyte score, and stage (Fig. 1). The demographic, clinical, and pathologic characteristics of each patient cohort is summarized in Table 1. Microsatellite status composition of patients with CL-High and CL-Low tumors was significantly different in the right-sided colon cancer (*p* < 0.0001) and rectal cancer (*p* = 0.0264) groups with more MSI-H patients among the CL-High patients at both locations (Table 1). Additionally, cytotoxic lymphocyte scores correlated significantly with pathologic tumor stage in right-sided colon cancer (*p* = 0.0278) and rectal cancer (*p* = 0.0279) patients, but not in left-sided colon cancer patients (Table 1). This analysis demonstrates that there are significant differences based on tumor location, suggesting that this variable is an important consideration when analyzing patient data [15, 23].

**Table 1:** Patient Characteristics

Characteristic	High	Low	*P* value
Gender, n			
RSC	156	96	0.3637
Male	77	54	
Female	77	42	
LSC	68	106	0.1231
Male	41	51	
Female	27	55	
REC	63	91	0.5118
Male	37	48	
Female	26	43	
MS, n			
RSC	156	96	< 0.0001*
MSS/MSI-L	92	89	
MSI-H	64	7	
LSC	68	106	0.0771
MSS/MSI-L	64	105	
MSI-H	4	1	
REC	63	91	0.0264*
MSS/MSI-L	59	91	
MSI-H	4	0	
Age,Mean ± SD			
RSC	70 ± 13.69	67 ± 12.95	0.2222
LSC	66 ± 11.11	64 ± 13.07	0.4267
REC	64 ± 10.56	65 ± 12.32	0.81
Pathologic stage, n (%)			
RSC	156	96	0.0278*
I	33(21.2%)	11(11.5%)	
II	73(46.8%)	38(39.6%)	
III	38(24.4%)	32(33.3%)	
IV	12(7.7%)	15(15.6%)	
LSC	68	106	0.1374
I	12(17.6%)	17(16.0%)	
II	26(38.2%)	33(31.1%)	
III	23(33.8%)	30(28.3%)	
IV	7(10.3%)	26(24.5%)	
REC	63	91	
I	10(15.9%)	20(22.0%)	0.0279*
II	29(46.0%)	21(23.1%)	
III	17(27.0%)	33(36.3%)	
IV	7(11.1%)	17(18.7%)	

*RSC* Right-sided colon cancer, *LSC* Left-sided colon cancer, *REC* Rectal cancer, *MSS* Microsatellite stable, *MSI-L* Microsatellite instability-low, *MSI-H* Microsatellite instability-high

*Indicates statistically significant difference (*p* < 0.05)

### Differential gene expression analysis

For each cohort, we next performed RNA-seq differential gene expression analysis. Expression of 20,531 genes was determined for each tumor sample from the TCGA. Then gene expression was compared between patients with CL-High and CL-Low tumors in each cohort based on tumor location and stage using the edgeR package (analysis included in Additional file 2). In right-sided early, localized, and metastatic colon cancer patients, 1882, 1781, and 1054 differentially expressed genes were observed, respectively. In left-sided patients, 805, 925, and 1255 genes were differentially expressed in each stage. And in rectal cancer patients, 888, 1316 and 150 genes were differentially expressed at each stage (Fig. 1). In the left-sided group, differentially expressed genes were highest in the metastatic cohort; however, in right-sided and rectal cancer patients, the metastatic cohort had the lowest number of differentially expressed genes. This again suggests that both tumor location and stage are important considerations when analyzing alterations in gene expression. Differentially expressed genes found in the above analysis were subsequently imported into the Reactome Pathway Browser to determine involvement in immune related functional pathways (Fig. 1, [15]). Interestingly, despite significant variation in the number of differentially expressed genes, the ratio of genes associated with immune function was similar in all sites and stages.

### Pathway enrichment and survival analysis

**Fig. 2:**
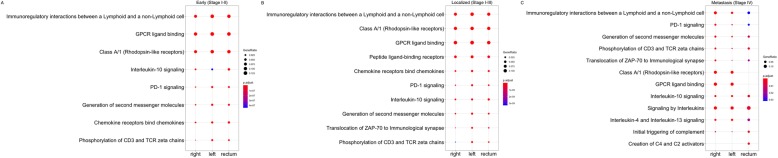
Pathway Enrichment analysis based on tumor location and stage. Pathway enrichment was ranked using a composite of the adjusted p value for false discovery rate (color) and gene ratio (size). **a** pathway enrichment for early stage CRC (Stage I and II) based on location; **b** pathway enrichment analysis for localized CRC (Stages I-III) based on location; **c** pathway enrichment analysis for metastatic stage CRC (Stage IV) based on location. This analysis showed that the “immunoregulatory interactions between a lymphoid and a non-lymphoid cell” was the most highly enriched pathway in early and local patients at all sites. Additionally, this was the most highly enriched pathway in patients with metastatic right-sided cancer. This pathway was also among the top pathways enriched among patients with metastatic left-sided colon cancer and rectal cancer. (Input data included in Additional file 2)

To further determine whether there were overlapping pathways associated with cytotoxic lymphocyte infiltration in colorectal cancer, we then compared the Reactome pathway enrichment analysis at each location based on stage. Using the *p* value adjusted for false discovery rate and the ratio of differentially expressed genes in each pathway, we found that the “immunoregulatory interactions between a lymphoid and a non-lymphoid cell” was the most highly enriched pathway in early and local patients at all tumor locations. Additionally, this was the most highly enriched pathway in patients with metastatic right-sided cancer. This pathway was also among the top pathways enriched among patients with metastatic left-sided colon cancer and rectal cancer (Fig. 2, data and analysis included in Additional file 2). This suggests that despite significant heterogeneity among subjects, redundant pathways of deregulation may be conserved across stage and location.

**Fig. 3:**
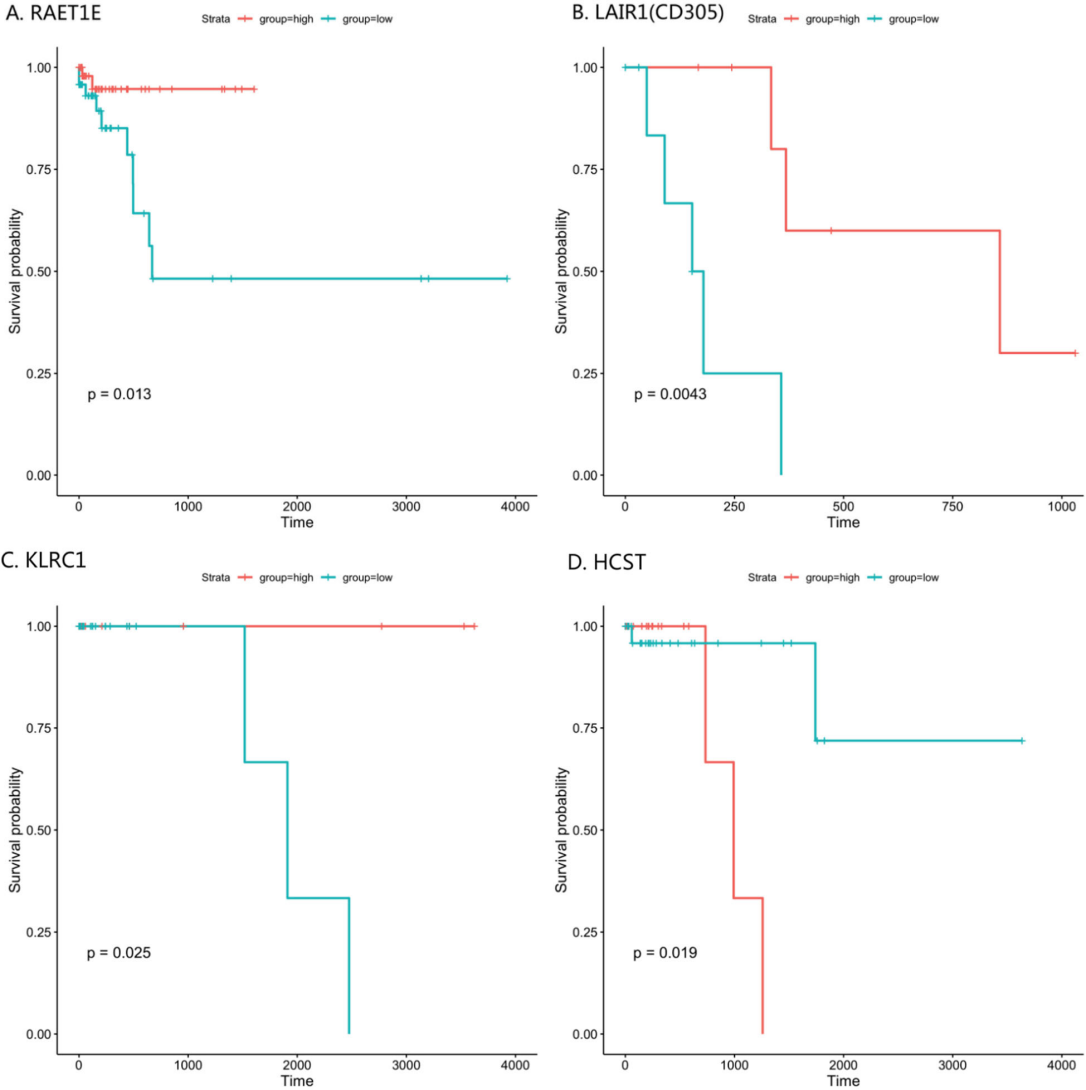
Representative survival curves based on tumor location and stage. Survival curves with the *p* values derived from Kaplan-Meier analysis. **a** RAET1E was positively associated with survival in right-sided colon cancer patients with high cytotoxic lymphocyte scores in early and localized stages; **b** LAIR1(CD305) was positively associated with survival in right-sided colon cancer patients with low cytotoxic lymphocyte scores in the metastatic stage; **c** KLRC1 was positively associated with survival in left-sided colon cancer patients with low cytotoxic lymphocyte scores in the early stage; **d** HCST was negatively associated with survival in rectal cancer patients with low cytotoxic lymphocyte scores in the localized group

To further understand the potential for targetable genes within this pathway, we then took differentially expressed genes in the “immunoregulatory interactions between a lymphoid and non-lymphoid cell” pathway and performed a survival analysis using the Kaplan-Meier method and Cox-Proportional Hazards Model based on differentially expressed gene, location, cytotoxic lymphocyte score, and pathologic stage (Fig. 3, Table 2, remaining survival curves and analysis included in Additional file 2). There are a total of 297 genes included in this pathway, and we found 21 (7.1%) unique genes associated with survival in this pathway. As Fig. 1 demonstrates, the number of differentially expressed genes were variable with most genes associated with survival in the right-sided colon cancer group. Additionally, the positive and negative impact of differentially expressed genes on survival depended on cytotoxic lymphocyte abundance scores. The majority of genes associated with a positive impact on survival (**bold in** Table 2) were in the CL-Low group whereas the majority of genes with a negative impact on survival (*italicized in* Table 2) were found in the CL-High group. In CL-High right-sided colon cancer patients with metastatic disease, all differentially expressed genes had a negative impact on survival; however, all immune functional differentially expressed genes in the CL-Low group had a positive impact on survival. Often, patients with rectal cancer and left-sided cancer are considered to have similar disease biologically. While we found few differentially expressed genes in this pathway associated with survival in the left-sided and rectal cancer groups, differentially expressed genes in the left-sided colon cancer group primarily had a positive impact on survival with the converse being true in patients in the rectal cancer group. Together this data demonstrates that even within conserved immune pathways, there is significant heterogeneity in the impact on patient survival. This further suggests the importance of a patient centered approach for the application of immune based therapy in colorectal cancer.

**Table 2:** Differentially expressed genes in the “Immunoregulatory interactions between a lymphoid and a non-lymphoid cell” pathway associated with survival grouped by pathologic stage, cytotoxic lymphocyte (CL) status, and anatomical location

Immunoregulatory interactions between a Lymphoid and a non-Lymphoid cell				
Location	Stages	CLs Status	Gene Symbol	*P*-value
Right	Early (I,II)	High	**RAET1E**	0.033
			*LILRA1*	0.031
			*CD33*	0.018
		Low	N/A	
	Localized (I,II,III)	High	**RAET1E**	0.013
			*LILRA1*	0.0087
			*LILRA4*	0.042
			*CD33*	0.017
		Low	*KLRC1*	0.027
	Metastasis (IV)	High	*MADCAM1*	0.046
			*FCGR2B(CD32)*	0.046
			*SIGLEC8(CD329)*	0.046
			*CLEC4G*	0.046
		Low	**CD96**	0.027
			**LILRB1**	0.015
			**ITGB2(CD18)**	0.033
			**CD40LG**	0.029
			**C3**	0.018
			**SIGLEC9**	0.0071
			**SH2D1A**	0.027
			**LAIR1(CD305)**	0.0043
			**CD300A**	0.0043
Left	Early	High	N/A	
		Low	**KLRC1**	0.025
	Localized	High	N/A	
		Low	N/A	
	Metastasis	High	N/A	
		Low	**CLEC2B**	0.046
Rectum	Early	High	N/A	
		Low	*CLEC4G*	0.046
	Localized	High	N/A	
		Low	*HCST*	0.019
			*FCGR1A(CD64)*	0.039
			*CLEC4G*	0.022
	Metastasis	High	N/A	
		Low	N/A	

*CLs* Cytotoxic lymphocytes; gene symbols with bold font indicate positive impact on patient’s survival, gene symbols with italic font indicate negative impact on patient’s survival

## Discussion

Cytotoxic lymphocyte infiltration is critical for response to immune based therapy [6] and has been shown to predict survival and treatment response in colorectal cancer [4, 5]. A better understanding of potential targets is critical for the improvement of immune based therapy in colorectal cancer as currently utilized therapy is not effective in the majority of patients. Therefore, in this study we have combined publicly available data resources with computational methods to focus on genes that may have an impact both on tumor associated cytotoxic lymphocytes and survival [4]. Comparing patients with high and low cytotoxic lymphocyte abundance scores, we found many differentially expressed genes at all tumor locations and stages. Unsurprisingly, the group with the highest number of immune related differentially expressed genes was the right-sided colon cancer group. This may be a reflection of the higher number of MSI-H patients in this group, which is expected to have a higher mutation rate, and therefore, potentially more genes with altered expression.

To further define potential therapeutic targets, we then performed pathway enrichment analysis. In this analysis, we found the pathway, “immunoregulatory interactions between a lymphoid and non-lymphoid cell”, was among the most highly enriched and altered in all sites and stages. A few pathways were occasionally more highly enriched, however were not affected at all sites or stages, therefore, we chose to focus on this pathway. The “immunoregulatory interactions between a lymphoid and non-lymphoid cell” pathway involves a number of cell surface signaling pathways that are involved in the regulation of anti-tumor immunity [20]. After performing survival analyses using the differentially expressed genes from this pathway, we found the majority of genes affecting survival were in the right-sided patient group consistent with the differential gene expression analysis. Patients with right-sided colon cancer have significantly worse survival than other tumor locations at all stages, and right-sided colon cancer patients with metastatic disease demonstrate poorer survival with current chemotherapy regimens. This group, therefore, likely represents the group with the most important need for new therapeutic options [15, 23]. There was, however, a clear dichotomy between patients with high and low cytotoxic lymphocyte abundance scores. Nearly all genes affecting survival found in the CL-Low patients had a positive impact, whereas nearly all genes affecting survival in the CL-High patients had a negative impact. This is not entirely unsuspected given we know that tumors attempt to evade anti-tumor immunity through various mechanisms [16]. Other groups have also demonstrated this in the context of MSI-H colorectal cancer, noting a significant upregulation of multiple negative regulators of immunity in these patients [9, 10]. This data further underscores the need to develop and test new immune based therapy in patients with colorectal cancer tailored to patient specific factors.

In our survival analysis, we identified several potential targets for combination therapy. CD40L is a cell surface marker expressed on activated T cells that promotes maturation of antigen presenting cells, upregulating co-stimulatory molecules and activating antigen presentation machinery, and may represent the most attractive target identified in this work. In our analysis, this gene demonstrated a positive impact on survival in metastatic patients with low cytotoxic lymphocyte abundance scores. In preclinical models, CD40 agonists have demonstrated a significant ability to activate anti-cancer immunity, overcome immune checkpoint inhibition resistance, and work in concert with other immune based treatments [24]. Currently, a number of clinical trials are open studying these drugs in combination with other immune based treatments; however, none are specifically directed at colorectal cancer patients. The fact that there are drugs available targeting this interaction may lend itself to rapid translation in these patients. Additionally, we found potentially attractive targets in CD96 and CD18 (ITGB2), each of which has demonstrated some significant impact on anti-tumor immunity in pre-clinical studies with the potential for translation in the future [25–27].

One limitation of this study is related to patient numbers and clinical data available, as with many database studies. Due to patient numbers, we included patients in Stage I and II in the analysis for both “early” and “local” disease. This was done to increase patient numbers assigned to each group and improve our analysis. Based on our results, we felt this helped to support findings in the “early” stage patients as the Stage III patients contributed 40–60% of “local” patients depending on disease location. Another important potential confounding factor, however, is significant heterogeneity in therapy, most notably in patients with metastatic disease (Stage IV). These were real world patients not treated on specific study protocols, so this heterogeneity in treatment may represent an impactful difference. Additionally, the patients included in this study did not receive immune based therapy, so the impact of cytotoxic lymphocyte infiltration on response to immune based treatments cannot be directly assessed. However, a number of studies have previously shown that cytotoxic lymphocyte infiltration in colorectal cancer predicts survival and response to therapy [4, 5, 8, 10], therefore augmenting anti-tumor immunity is likely to be impactful when considering combination with conventional treatments such as chemotherapy, or immune based therapy alone. Recent studies in cancer therapy have also begun to understand that the immune response is critical to the efficacy of chemotherapy and radiotherapy, further highlighting the need to understand altered immune pathways in cancer [28]. Despite these limitations, resources such as the TCGA, when combined with informatics-based analysis, yield highly impactful results that can be used to develop future human studies and inform translational pre-clinical studies. The goals of precision-based oncology will be best met by combining studies of all types to select both the best therapy for each patient, as well as the best patient for each therapy.

## Conclusion

In this study, we integrate comprehensive RNA-seq data, clinical and pathologic data, and cytotoxic lymphocyte scores to determine pathways associated with immune response and survival in patients with colorectal cancer. We identified one pathway, “immunoregulatory interactions between a lymphoid and non-lymphoid cell”, that was highly enriched and included in all tumor locations and stages. We then found specific genes associated with survival, primarily in patients with the worst survival, those with metastatic right-sided colon cancer, that may be targeted to improve therapy. Future studies will focus on further exploration of immune pathway interactions using multi-omics analysis in humans, and mechanistic studies of T lymphocyte recruitment and activation in murine models of colorectal cancer.

## Supplementary information

**Additional file 1: Figure S1.** Comparison of cytotoxic lymphocyte scores in High (CL-high) and Low (CL-low) patients. ****p < 0.0001. **Table S1.** Distribution of cytotoxic lymphocyte abundance scores including median with 95% CI and quartiles for the entire patient cohort.

**Additional file 2.** This data file includes the results of all differentially expressed genes when comparing each tumor location and stage. This file also includes all results from the pathway enrichment analysis that are included in the visualization (**Figure 2**). Additionally, the survival analysis from all genes with a significant impact on survival is included in this file.

## Data Availability

Full access to source data from the TCGA via FireCloud requires permission through dbgap. An application for full access to the TCGA data may be submitted here: https://dbgap.ncbi.nlm.nih.gov/aa/wga.cgi?page=login. All methods and R packages used in the preparation of this manuscript are available publicly (see below). For further information requests, please contact the corresponding author. TCGA data: https://firecloud.terra.bio/#workspaces/broad-firecloud-tcga/TCGA_COAD_ControlledAccess_V1-0_DATA https://firecloud.terra.bio/#workspaces/broad-firecloud-tcga/TCGA_READ_ControlledAccess_V1-0_DATA R package: MCP-counter https://github.com/ebecht/MCPcounter edgeR https://bioconductor.org/packages/release/bioc/html/edgeR.html clusterProfiler https://bioconductor.org/packages/release/bioc/html/clusterProfiler.html DOSE https://bioconductor.org/packages/release/bioc/html/DOSE.html ReactomePA https://bioconductor.org/packages/release/bioc/html/ReactomePA.html Survminer https://cran.r-project.org/web/packages/survminer/index.html

## Abbreviations

CGP: Comprehensive genomic profiling
CL: Cytotoxic lymphocytes
CL-High: Cytotoxic lymphocyte-high
CL-Low: Cytotoxic lymphocyte-low
COAD: Colon adenocarcinoma
CRC: Colorectal cancer
DEG: Differentially expressed genes
FDA: Food and Drug Administration
FDR: False discovery rate
ICI: Immune checkpoint inhibition
LSC: Left-sided colon cancer
MCP: Microenvironment cell populations
mRNA: Messenger ribonucleic acid
MS: Microsatellite stability
MSI-H: Microsatellite instability-high
MSI-L: Microsatellite instability-low
MSS: Microsatellite stable
OS: Overall survival
READ: Rectal adenocarcinoma
REC: Rectal cancer
RNA-seq: Ribonucleic acid sequencing
RSC: Right-sided colon cancer
RSEM: RNA-Seq by Expectation Maximization
SD: Standard deviation
TCGA: The Cancer Genome Atlas
TMB: Tumor mutational burden
