# Supplementary material for: Immunogenomic pathways associated with cytotoxic lymphocyte infiltration and survival in colorectal cancer
Source: BMC Cancer. 2020 Feb 14;20:124. doi: 10.1186/s12885-020-6513-4 (PMC7023815; doi:10.1186/s12885-020-6513-4)
Supplement: Supplementary file 1 — Additional file 1: Figure S1. Comparison of cytotoxic lymphocyte scores in High (CL-high) and Low (CL-low) patients. ****p < 0.0001. Table S1. Distribution of cytotoxic lymphocyte abundance scores including median with 95% CI and quartiles for the entire patient cohort. [file 12885_2020_6513_MOESM1_ESM.docx]

**Figure S1.** Comparison of cytotoxic lymphocyte scores in High (CL-high) and Low (CL-low) patients. ****p<0.0001.

**Table S1**. Distribution of cytotoxic lymphocyte abundance scores including median with 95% CI and quartiles for the entire patient cohort.

| Median and Quartiles | Score (95% CI) |
| --- | --- |
| Minimum | 1.785 |
| 25th Percentile | 13.95 |
| Median | **26.72 (24.1-30.1)** |
| 75th Percentile | 50.61 |
| Maximum | 546.3 |
