# Supplementary figures and images for: Immunogenomic pathways associated with cytotoxic lymphocyte infiltration and survival in colorectal cancer
Source: BMC Cancer. 2020 Feb 14;20:124. doi: 10.1186/s12885-020-6513-4 (PMC7023815; doi:10.1186/s12885-020-6513-4)

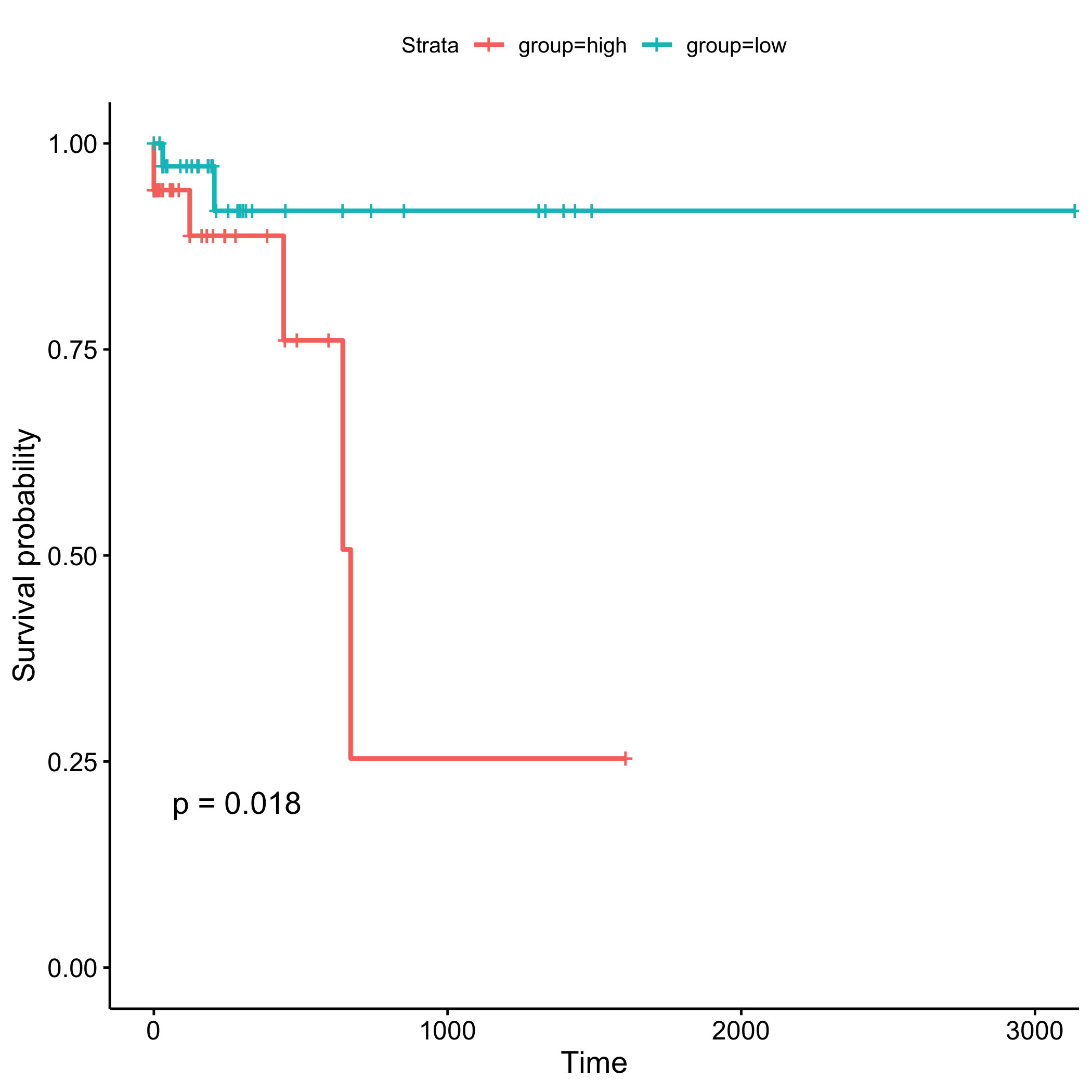

Supplement: Supplementary file 2 — Additional file 2. This data file includes the results of all differentially expressed genes when comparing each tumor location and stage. This file also includes all results from the pathway enrichment analysis that are included in the visualization (Figure 2). Additionally, the survival analysis from all genes with a significant impact on survival is included in this file. [file 12885_2020_6513_MOESM2_ESM.zip › 1.Right.early.high.CD33R4.png]

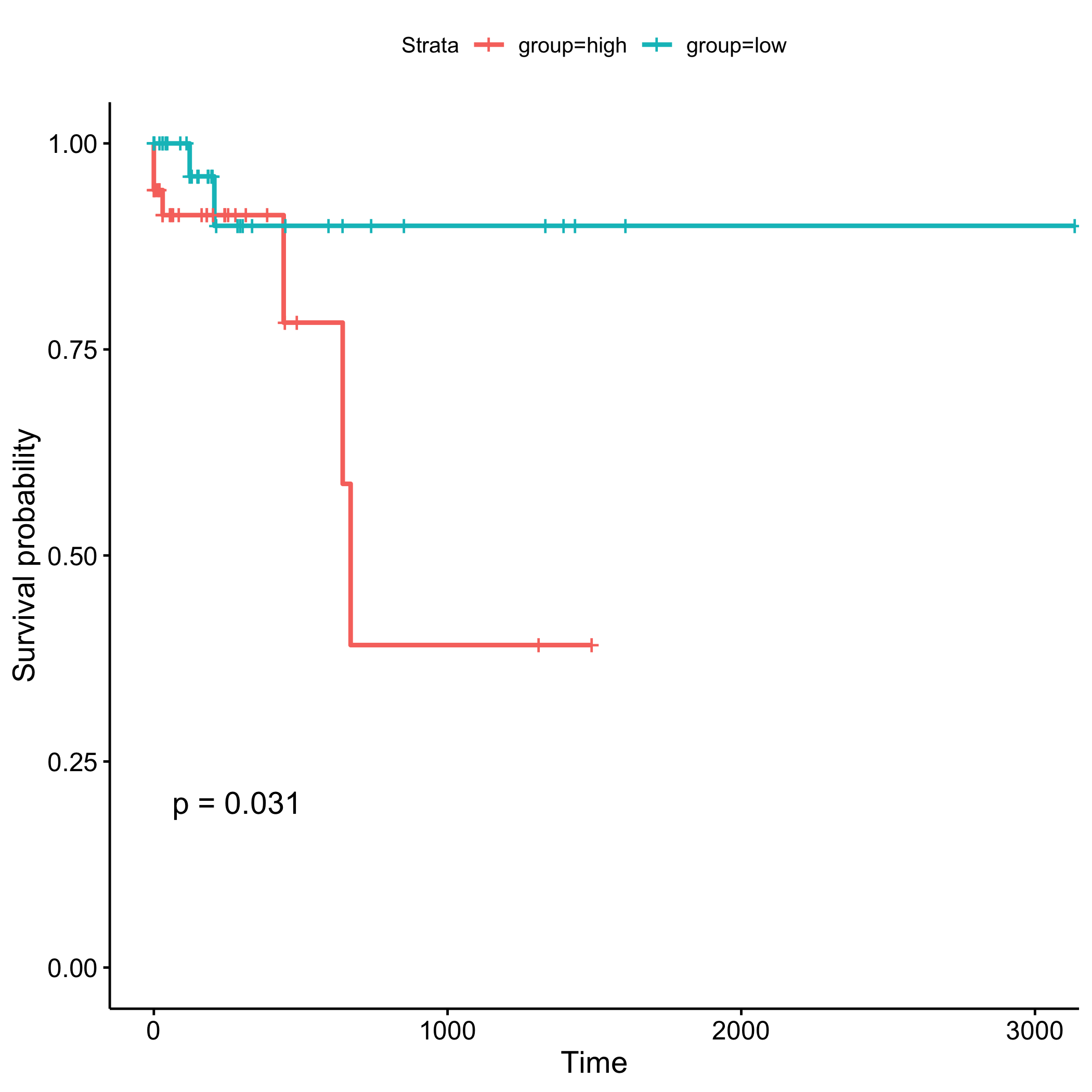

Supplement: Supplementary file 2 — Additional file 2. This data file includes the results of all differentially expressed genes when comparing each tumor location and stage. This file also includes all results from the pathway enrichment analysis that are included in the visualization (Figure 2). Additionally, the survival analysis from all genes with a significant impact on survival is included in this file. [file 12885_2020_6513_MOESM2_ESM.zip › 1.Right.early.high.LILRA1R4.png]

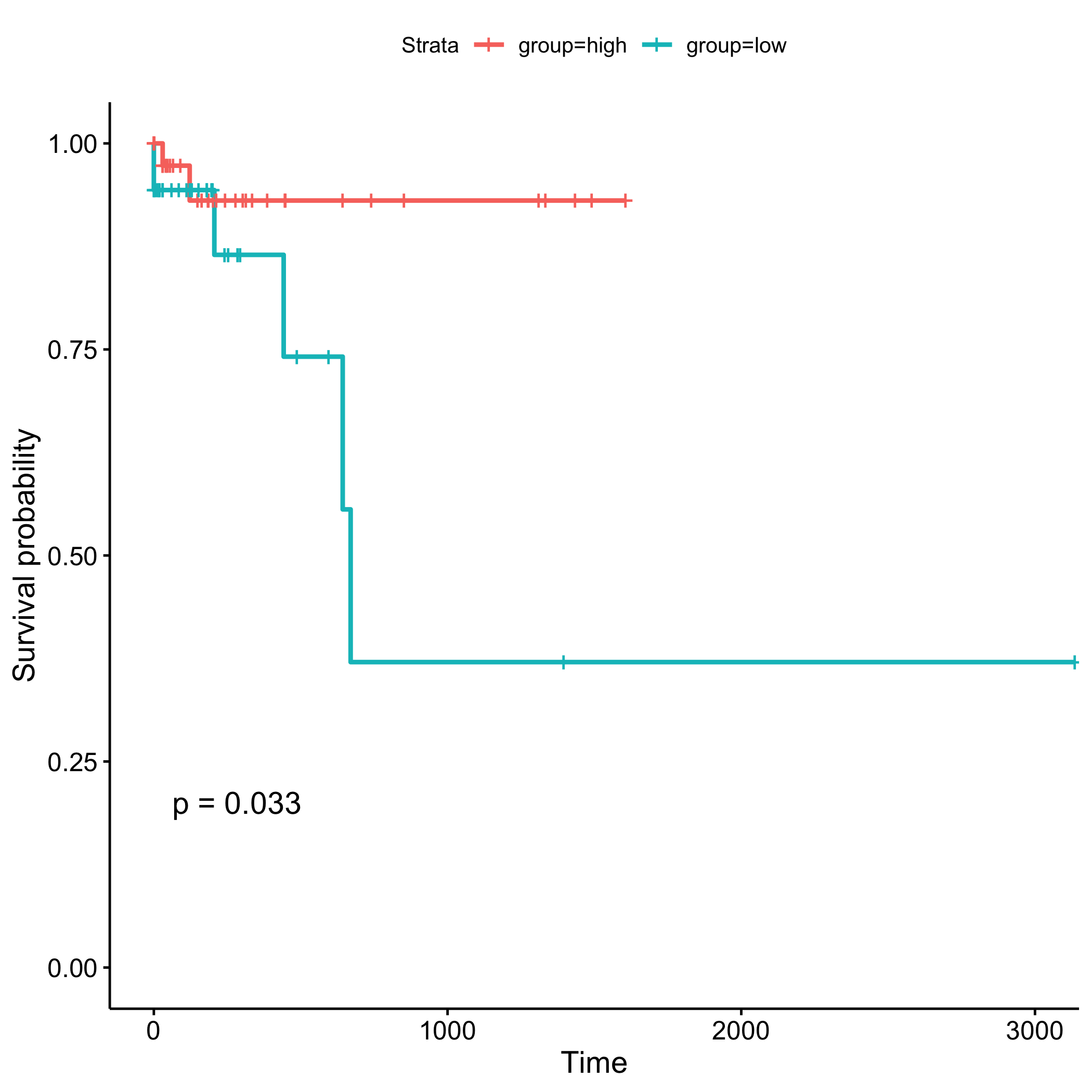

Supplement: Supplementary file 2 — Additional file 2. This data file includes the results of all differentially expressed genes when comparing each tumor location and stage. This file also includes all results from the pathway enrichment analysis that are included in the visualization (Figure 2). Additionally, the survival analysis from all genes with a significant impact on survival is included in this file. [file 12885_2020_6513_MOESM2_ESM.zip › 1.Right.early.high.RAET1ER4.png]

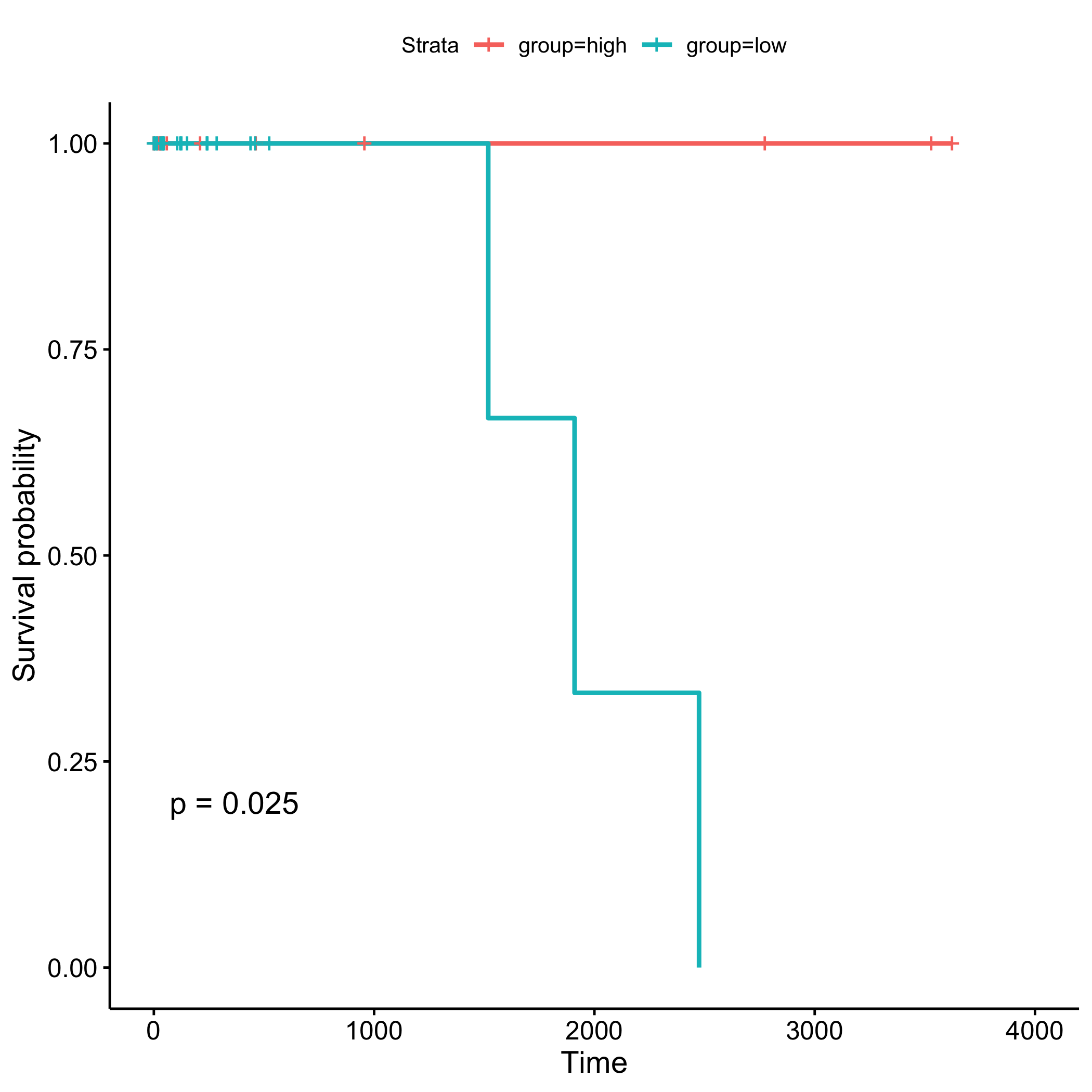

Supplement: Supplementary file 2 — Additional file 2. This data file includes the results of all differentially expressed genes when comparing each tumor location and stage. This file also includes all results from the pathway enrichment analysis that are included in the visualization (Figure 2). Additionally, the survival analysis from all genes with a significant impact on survival is included in this file. [file 12885_2020_6513_MOESM2_ESM.zip › 2.Left.early.low.survival.KLRC1R4.png]

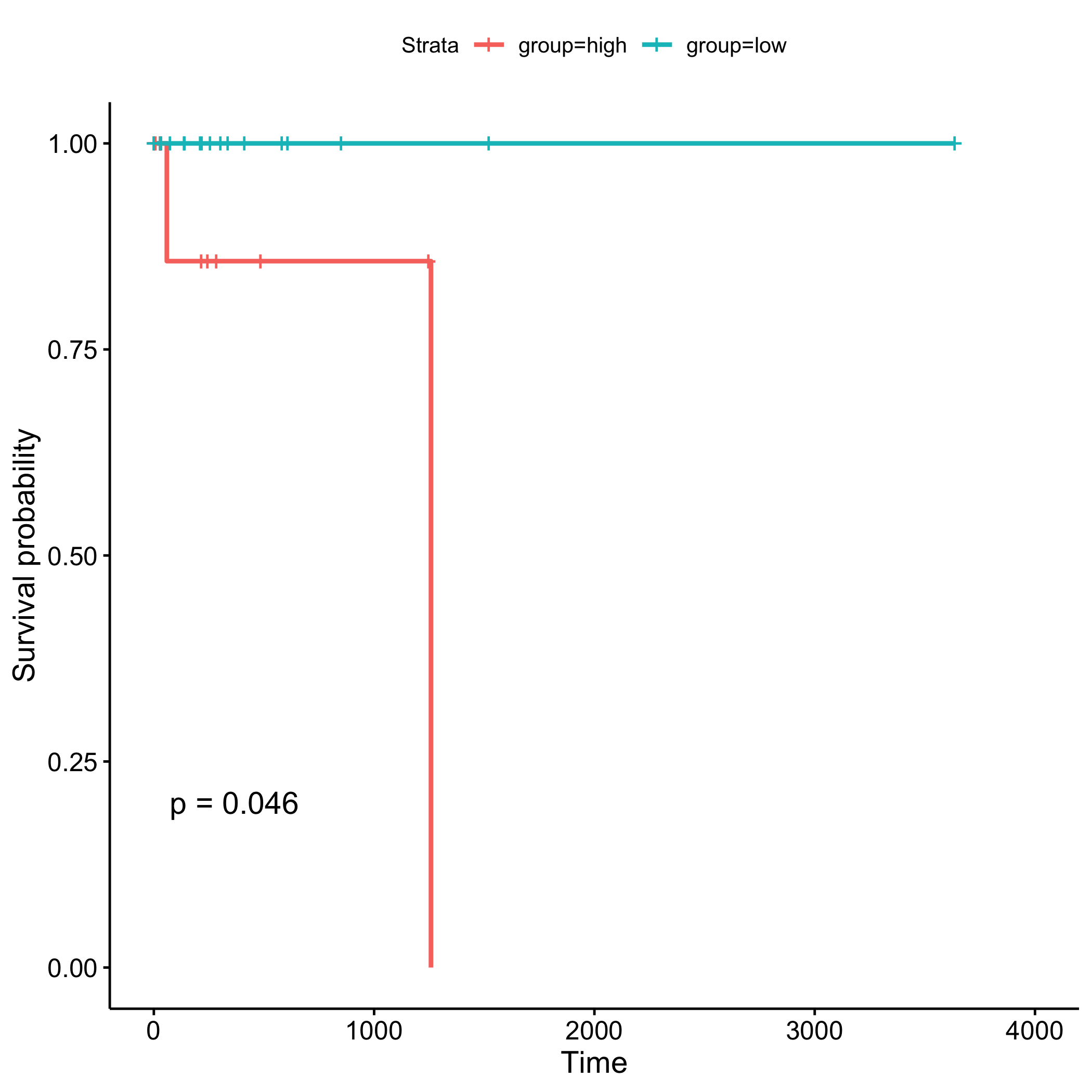

Supplement: Supplementary file 2 — Additional file 2. This data file includes the results of all differentially expressed genes when comparing each tumor location and stage. This file also includes all results from the pathway enrichment analysis that are included in the visualization (Figure 2). Additionally, the survival analysis from all genes with a significant impact on survival is included in this file. [file 12885_2020_6513_MOESM2_ESM.zip › 2.rectum.early.low.survival.CLEC4GR4.png]

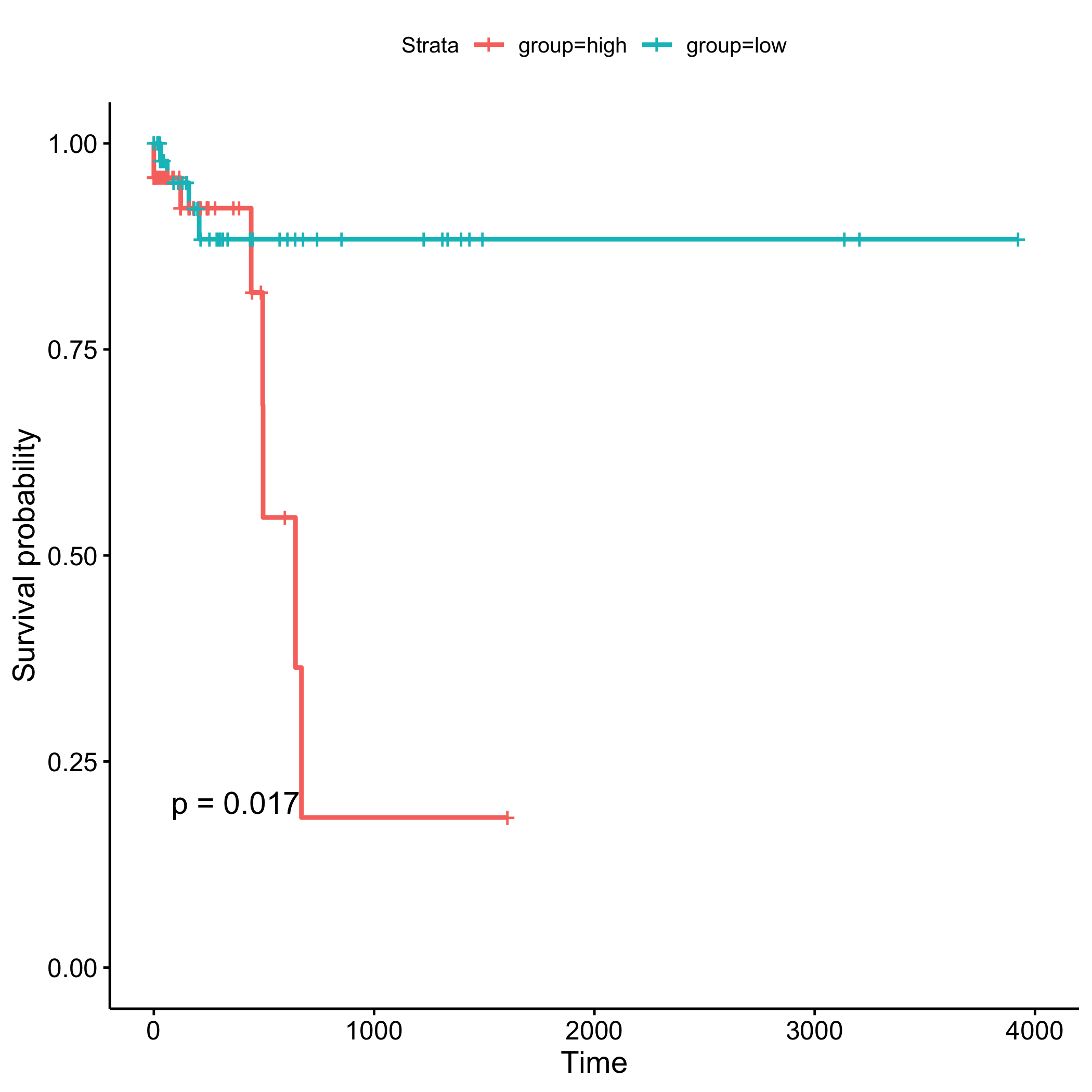

Supplement: Supplementary file 2 — Additional file 2. This data file includes the results of all differentially expressed genes when comparing each tumor location and stage. This file also includes all results from the pathway enrichment analysis that are included in the visualization (Figure 2). Additionally, the survival analysis from all genes with a significant impact on survival is included in this file. [file 12885_2020_6513_MOESM2_ESM.zip › 3.Right.loca.high.CD33R4.png]

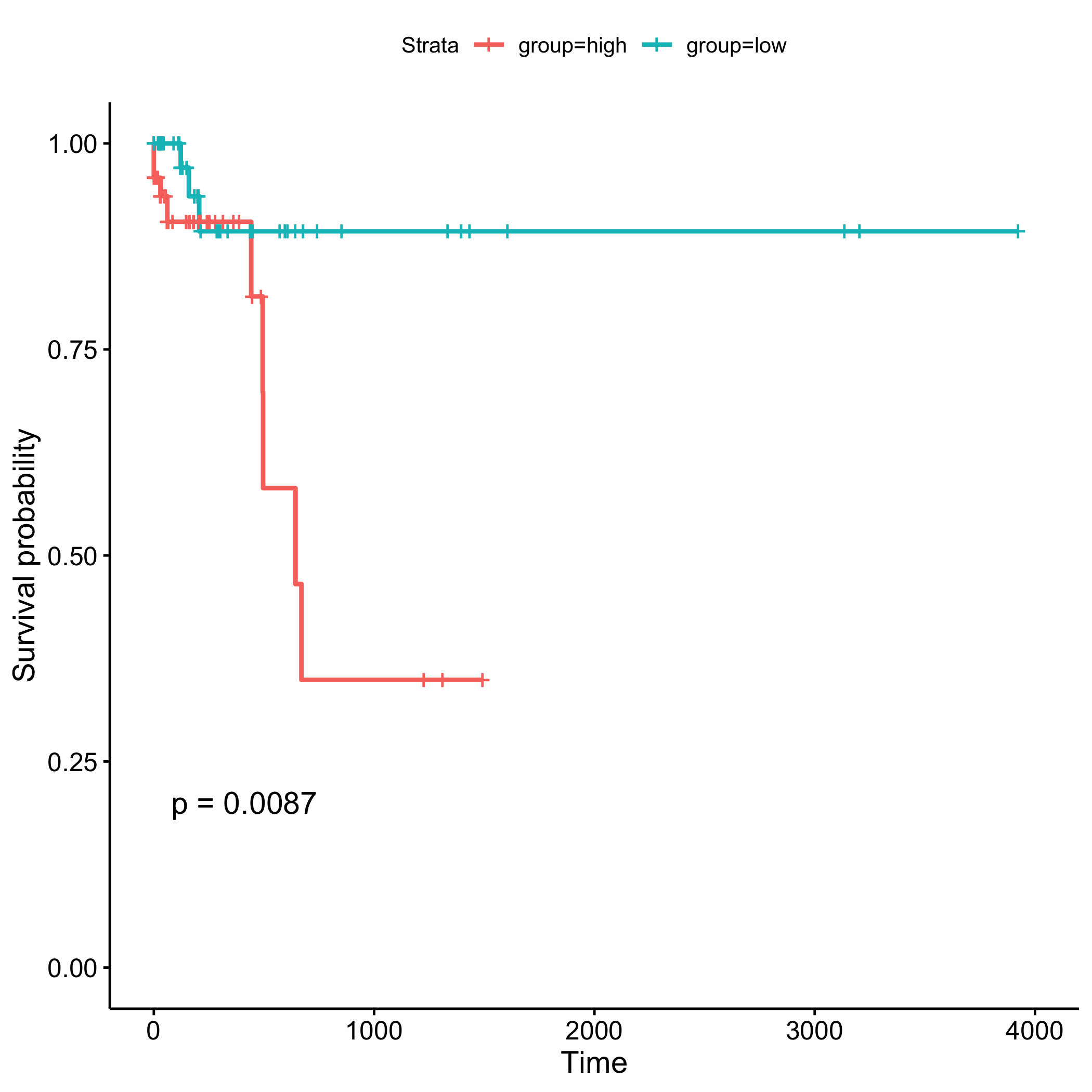

Supplement: Supplementary file 2 — Additional file 2. This data file includes the results of all differentially expressed genes when comparing each tumor location and stage. This file also includes all results from the pathway enrichment analysis that are included in the visualization (Figure 2). Additionally, the survival analysis from all genes with a significant impact on survival is included in this file. [file 12885_2020_6513_MOESM2_ESM.zip › 3.Right.loca.high.LILRA1R4.png]

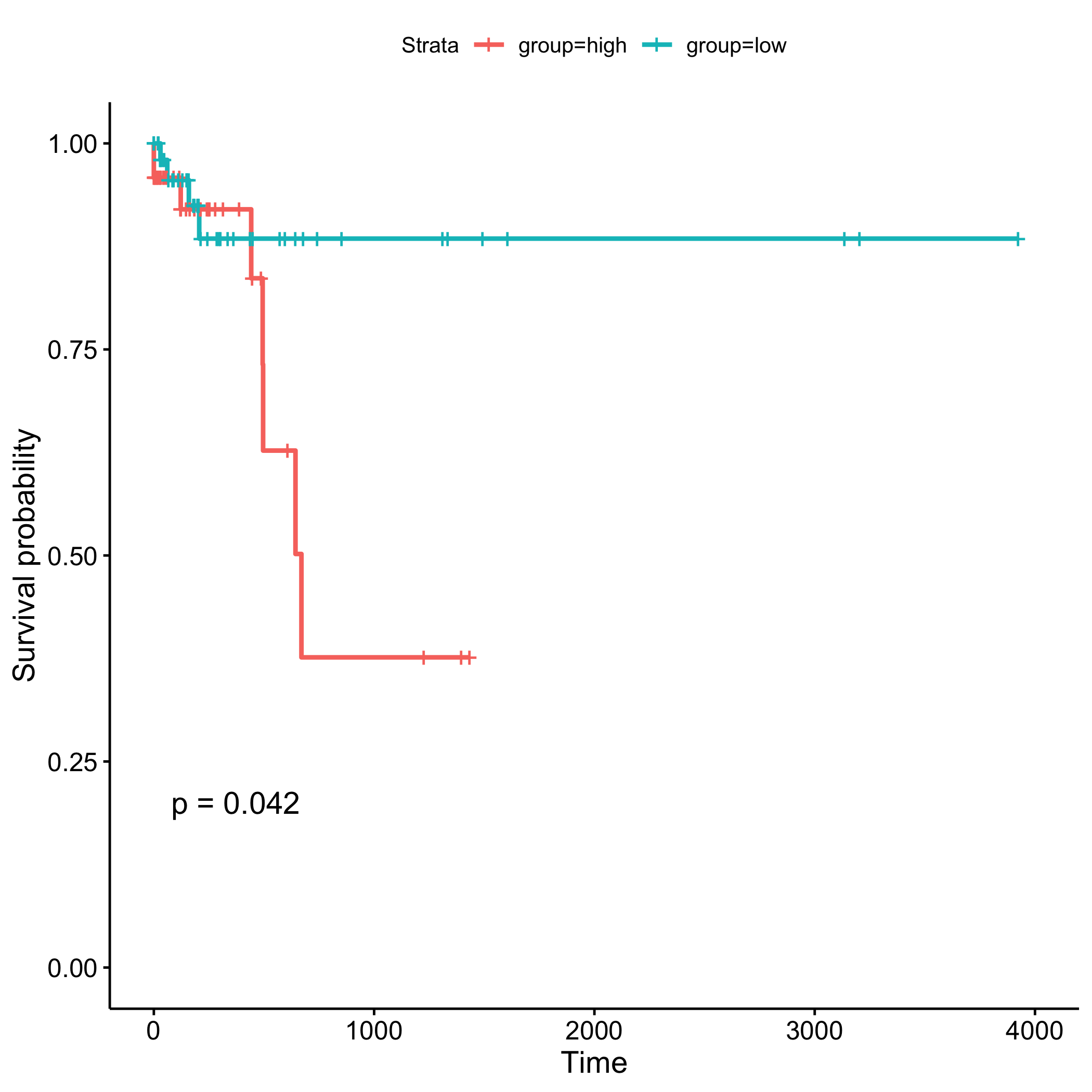

Supplement: Supplementary file 2 — Additional file 2. This data file includes the results of all differentially expressed genes when comparing each tumor location and stage. This file also includes all results from the pathway enrichment analysis that are included in the visualization (Figure 2). Additionally, the survival analysis from all genes with a significant impact on survival is included in this file. [file 12885_2020_6513_MOESM2_ESM.zip › 3.Right.loca.high.LILRA4R4.png]

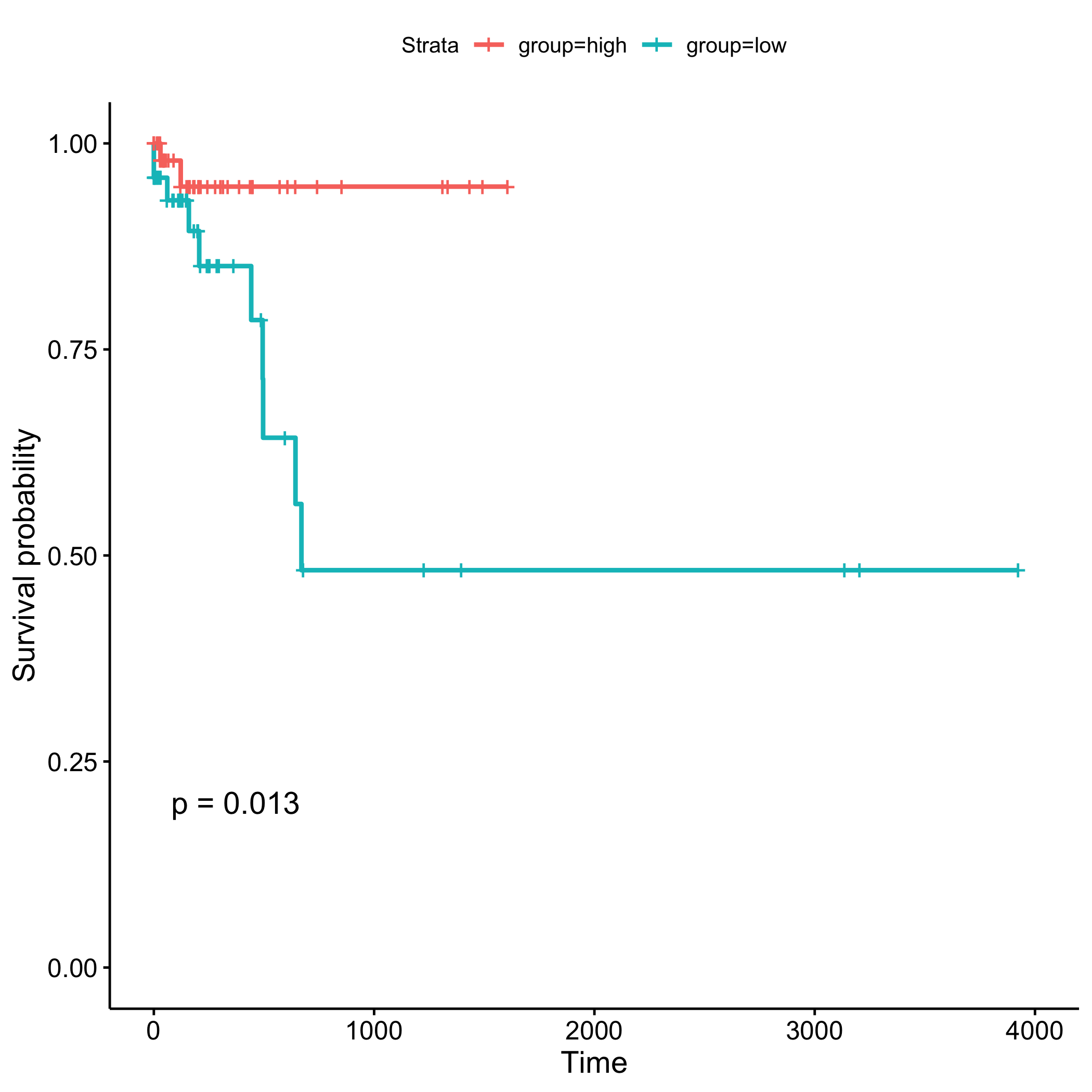

Supplement: Supplementary file 2 — Additional file 2. This data file includes the results of all differentially expressed genes when comparing each tumor location and stage. This file also includes all results from the pathway enrichment analysis that are included in the visualization (Figure 2). Additionally, the survival analysis from all genes with a significant impact on survival is included in this file. [file 12885_2020_6513_MOESM2_ESM.zip › 3.Right.loca.high.RAET1ER4.png]

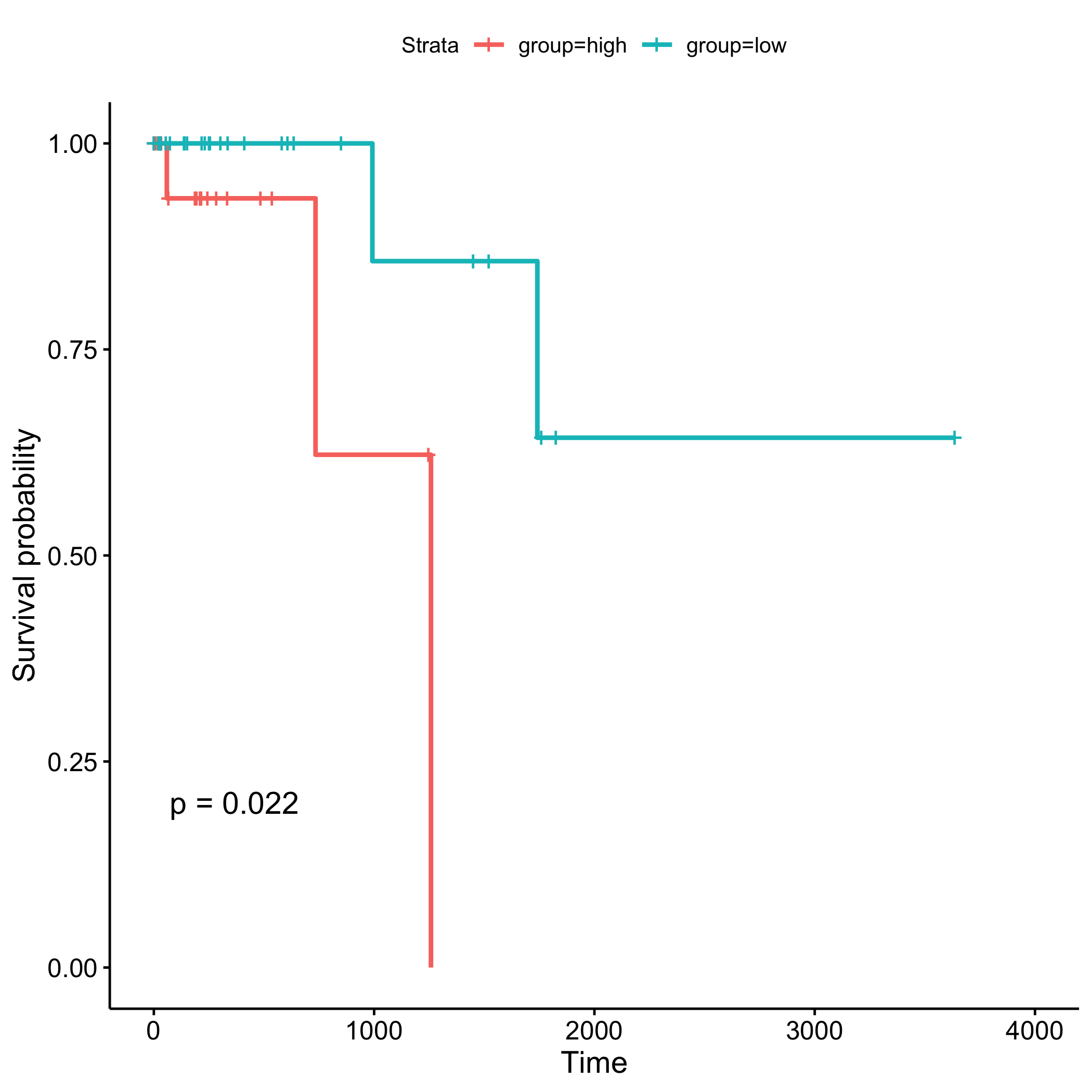

Supplement: Supplementary file 2 — Additional file 2. This data file includes the results of all differentially expressed genes when comparing each tumor location and stage. This file also includes all results from the pathway enrichment analysis that are included in the visualization (Figure 2). Additionally, the survival analysis from all genes with a significant impact on survival is included in this file. [file 12885_2020_6513_MOESM2_ESM.zip › 4.rectum.loca.low.survival.CLEC4GR4.png]

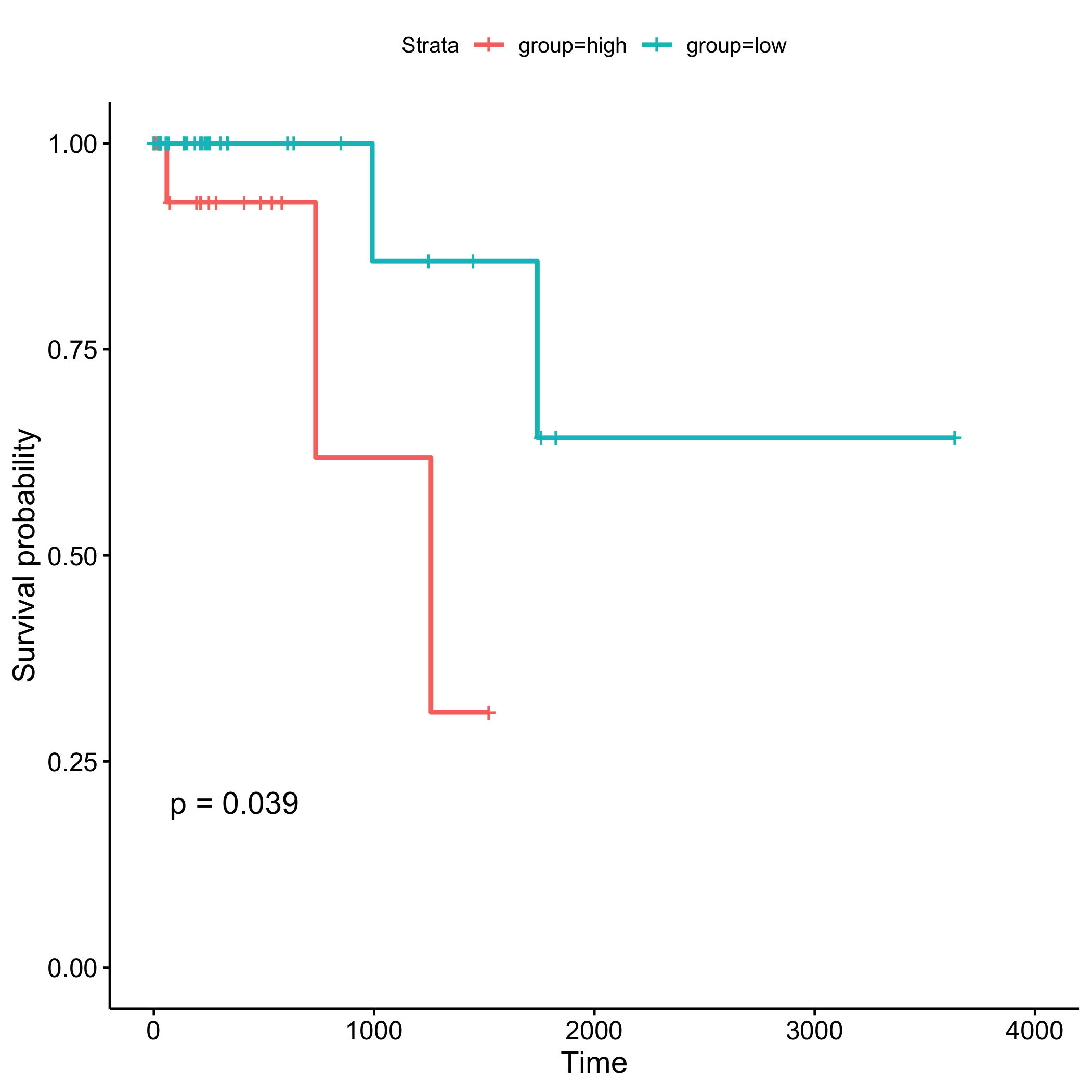

Supplement: Supplementary file 2 — Additional file 2. This data file includes the results of all differentially expressed genes when comparing each tumor location and stage. This file also includes all results from the pathway enrichment analysis that are included in the visualization (Figure 2). Additionally, the survival analysis from all genes with a significant impact on survival is included in this file. [file 12885_2020_6513_MOESM2_ESM.zip › 4.rectum.loca.low.survival.FCGR1AR4.png]

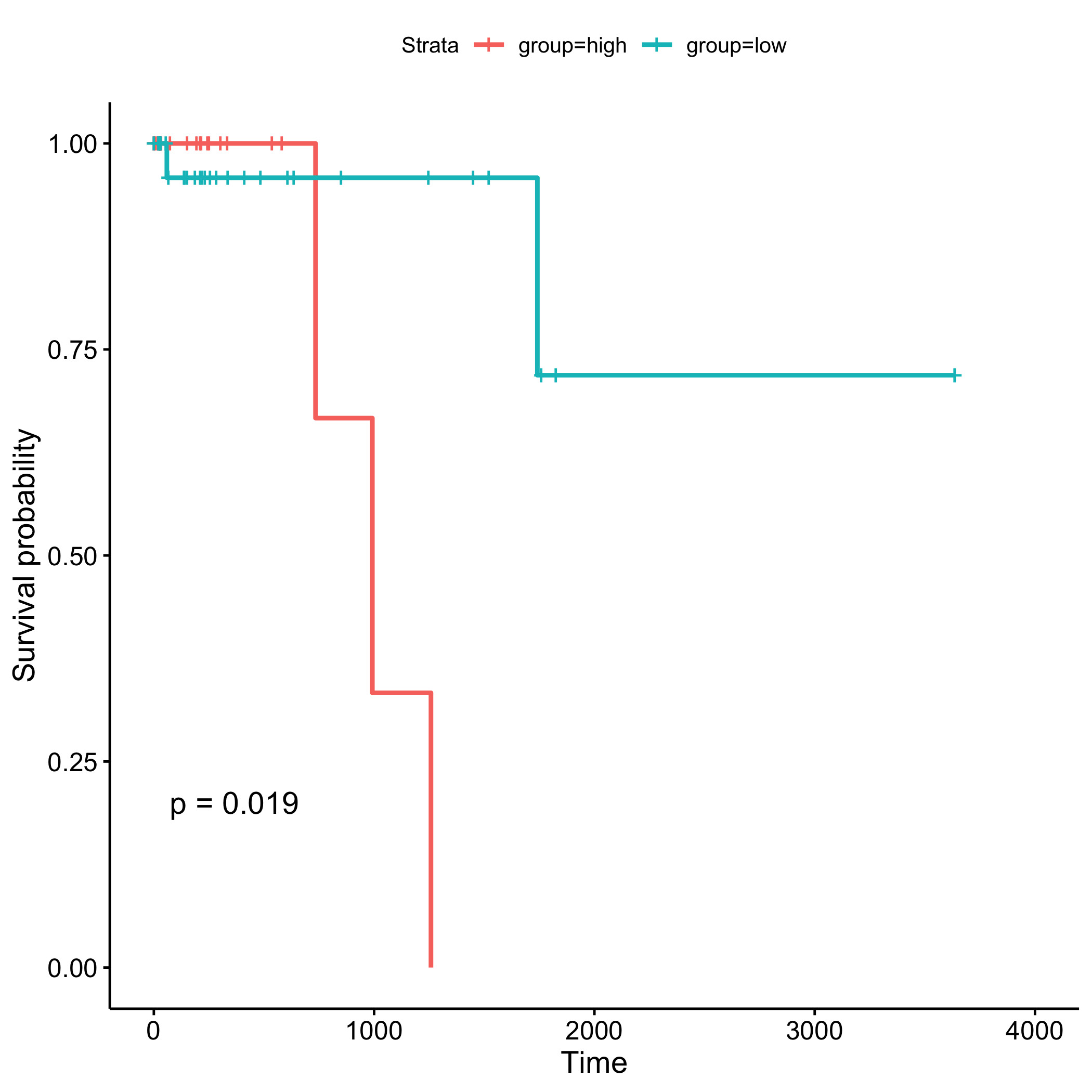

Supplement: Supplementary file 2 — Additional file 2. This data file includes the results of all differentially expressed genes when comparing each tumor location and stage. This file also includes all results from the pathway enrichment analysis that are included in the visualization (Figure 2). Additionally, the survival analysis from all genes with a significant impact on survival is included in this file. [file 12885_2020_6513_MOESM2_ESM.zip › 4.rectum.loca.low.survival.HCSTR4.png]

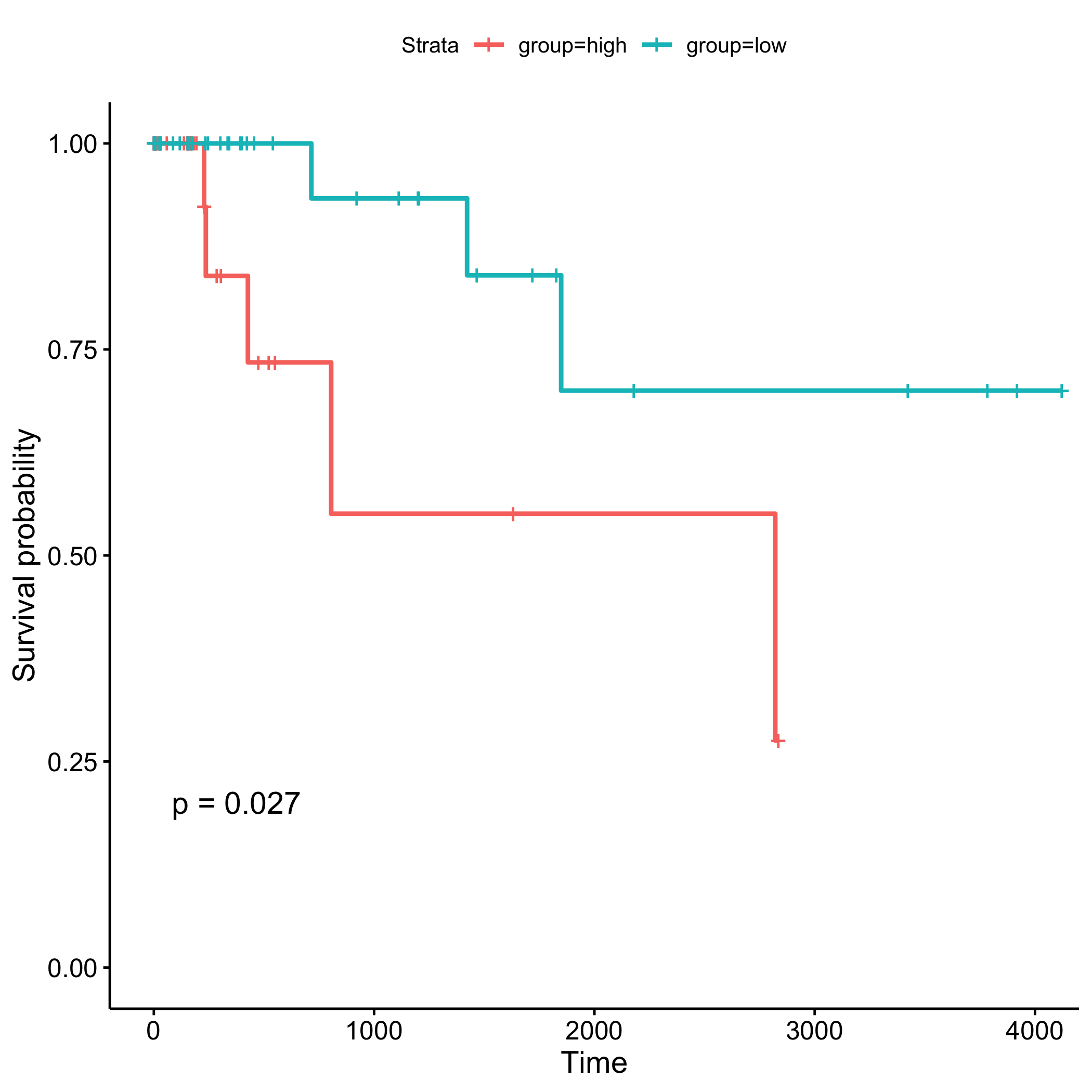

Supplement: Supplementary file 2 — Additional file 2. This data file includes the results of all differentially expressed genes when comparing each tumor location and stage. This file also includes all results from the pathway enrichment analysis that are included in the visualization (Figure 2). Additionally, the survival analysis from all genes with a significant impact on survival is included in this file. [file 12885_2020_6513_MOESM2_ESM.zip › 4.Right.loca.low.KLRC1R4.png]

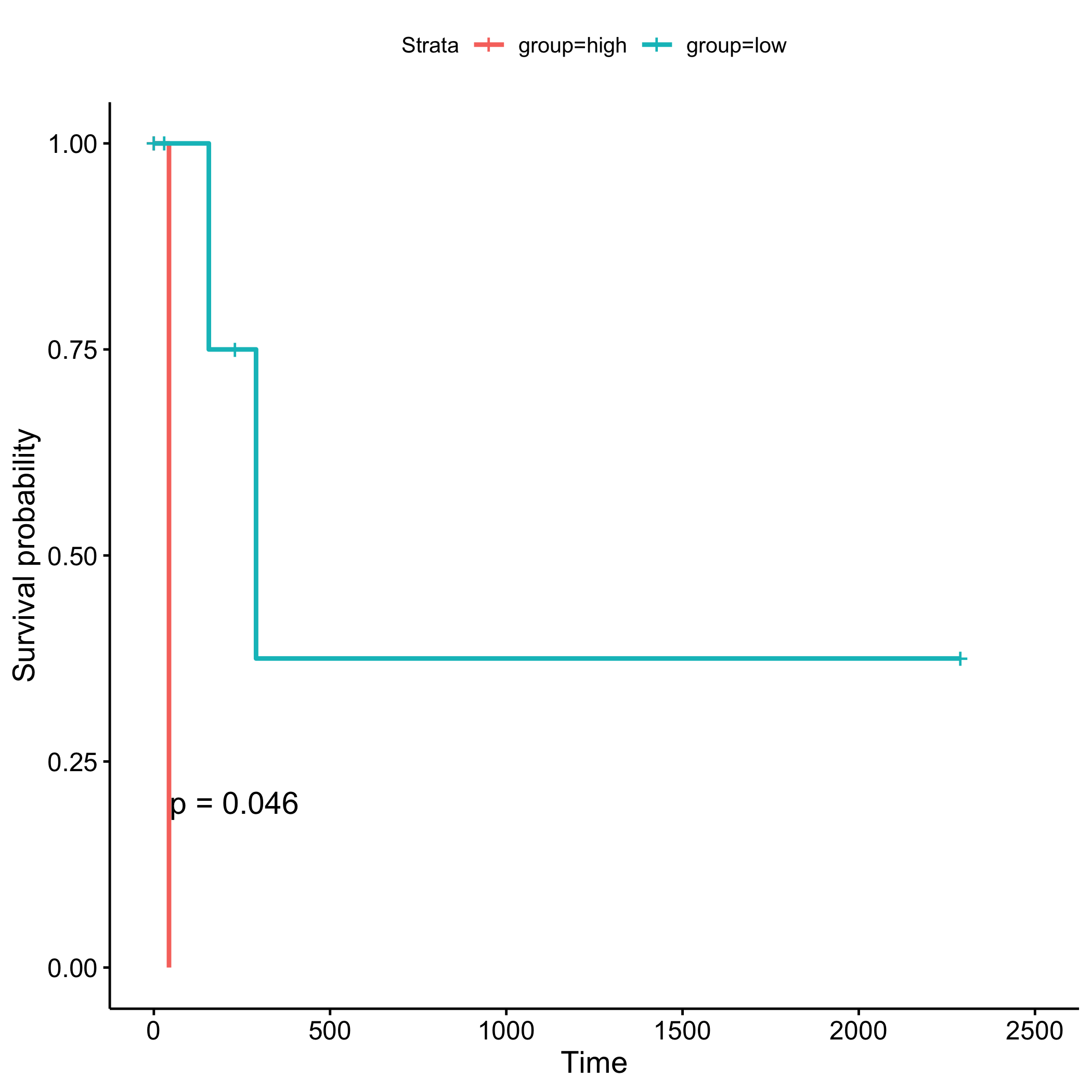

Supplement: Supplementary file 2 — Additional file 2. This data file includes the results of all differentially expressed genes when comparing each tumor location and stage. This file also includes all results from the pathway enrichment analysis that are included in the visualization (Figure 2). Additionally, the survival analysis from all genes with a significant impact on survival is included in this file. [file 12885_2020_6513_MOESM2_ESM.zip › 5.Right.meta.high.CD48R4.png]

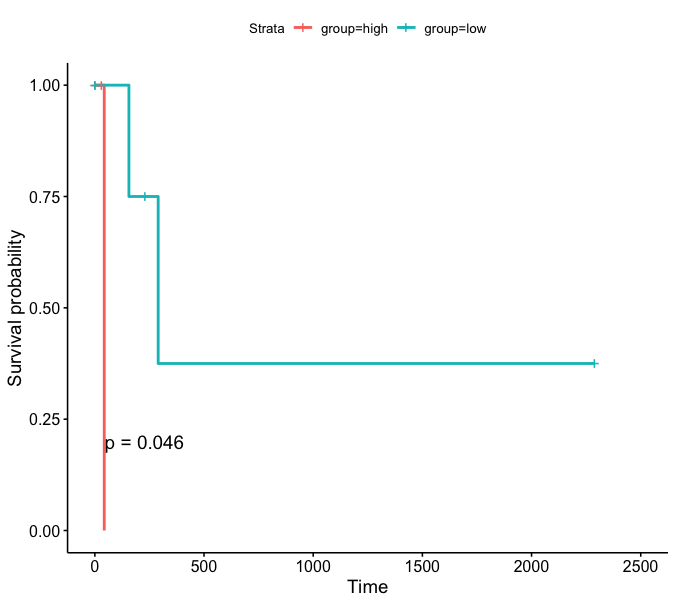

Supplement: Supplementary file 2 — Additional file 2. This data file includes the results of all differentially expressed genes when comparing each tumor location and stage. This file also includes all results from the pathway enrichment analysis that are included in the visualization (Figure 2). Additionally, the survival analysis from all genes with a significant impact on survival is included in this file. [file 12885_2020_6513_MOESM2_ESM.zip › 5.Right.meta.high.CLEC4GR4.png]

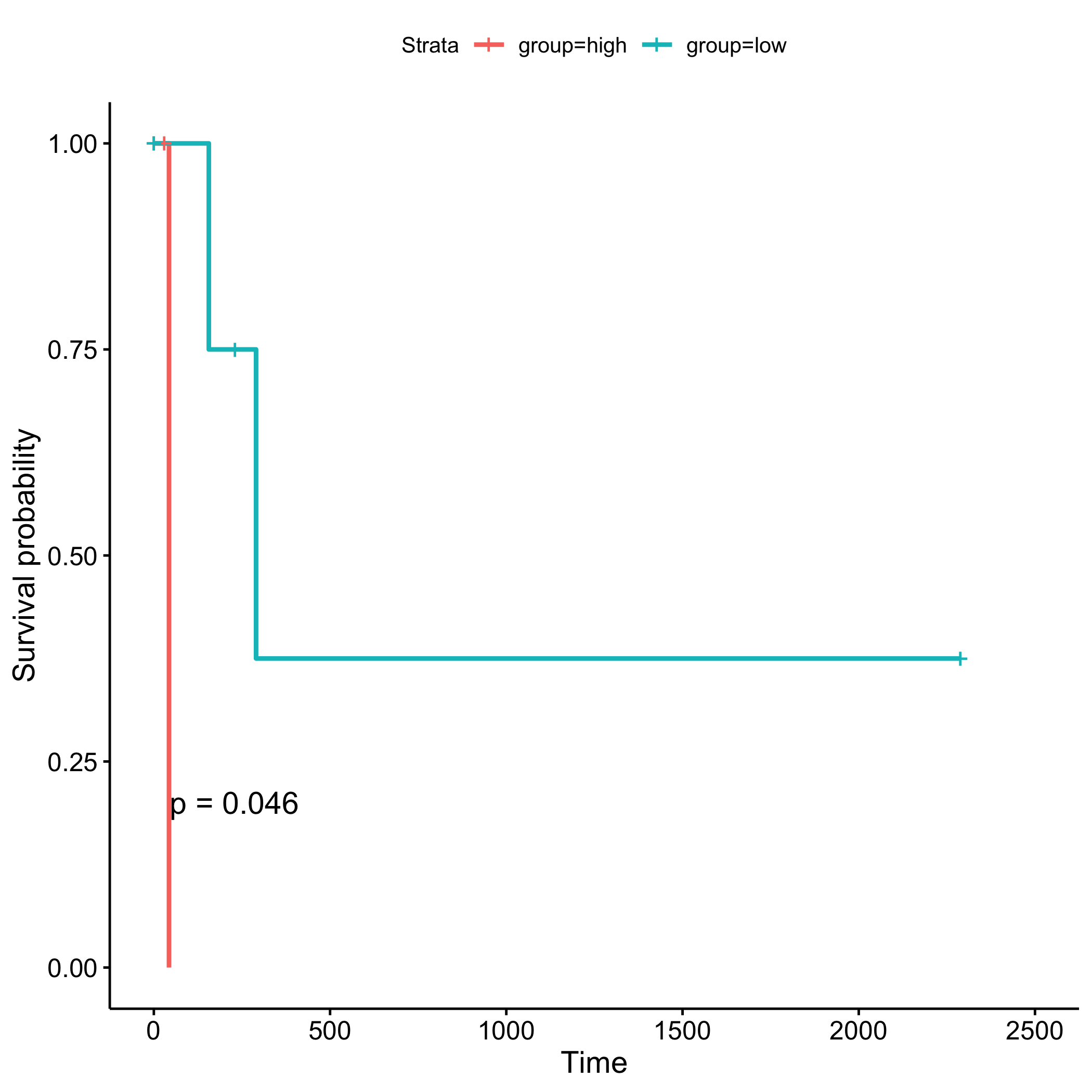

Supplement: Supplementary file 2 — Additional file 2. This data file includes the results of all differentially expressed genes when comparing each tumor location and stage. This file also includes all results from the pathway enrichment analysis that are included in the visualization (Figure 2). Additionally, the survival analysis from all genes with a significant impact on survival is included in this file. [file 12885_2020_6513_MOESM2_ESM.zip › 5.Right.meta.high.MADCAM1R4.png]

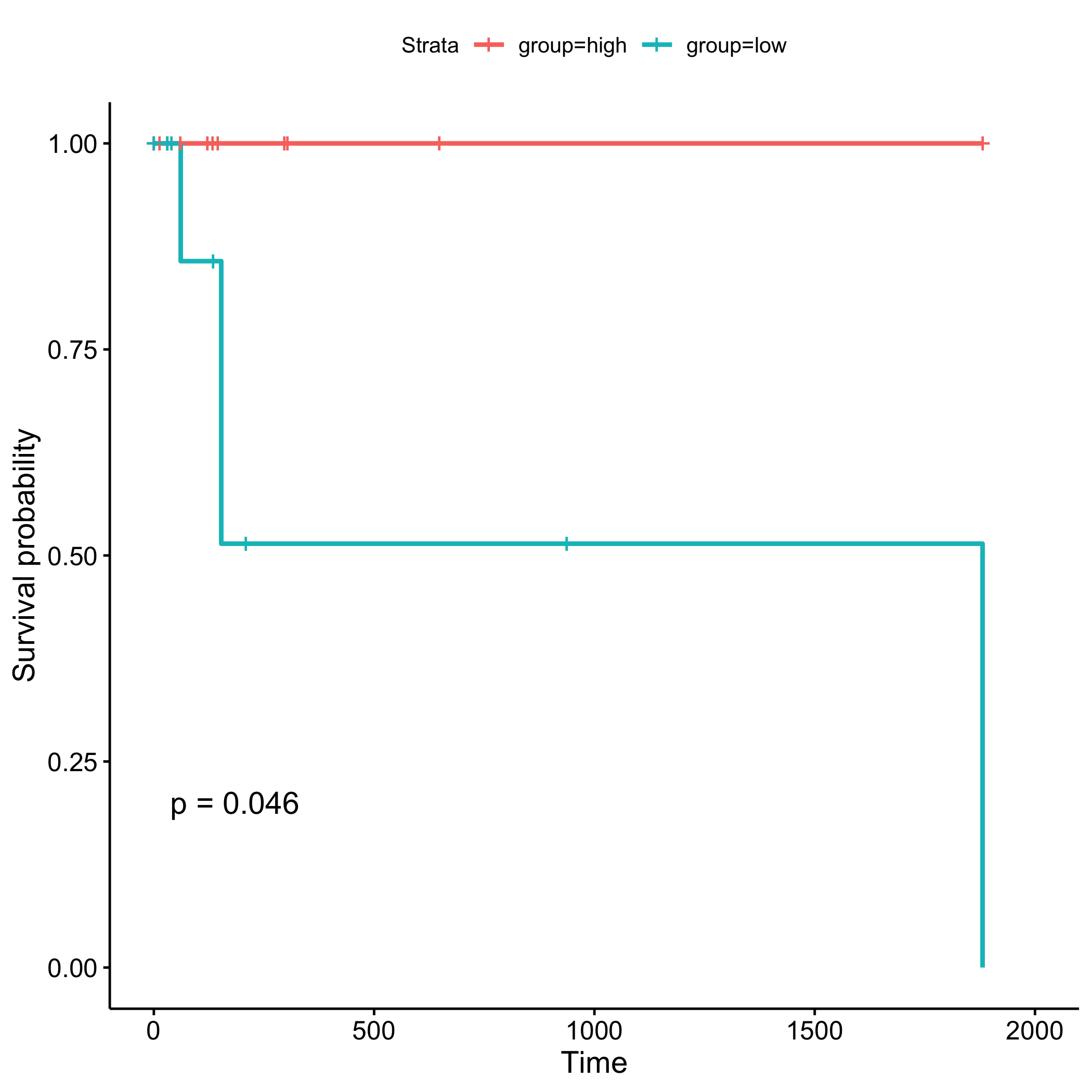

Supplement: Supplementary file 2 — Additional file 2. This data file includes the results of all differentially expressed genes when comparing each tumor location and stage. This file also includes all results from the pathway enrichment analysis that are included in the visualization (Figure 2). Additionally, the survival analysis from all genes with a significant impact on survival is included in this file. [file 12885_2020_6513_MOESM2_ESM.zip › 6.Left.meta.low.survival.CLEC2BR4.png]

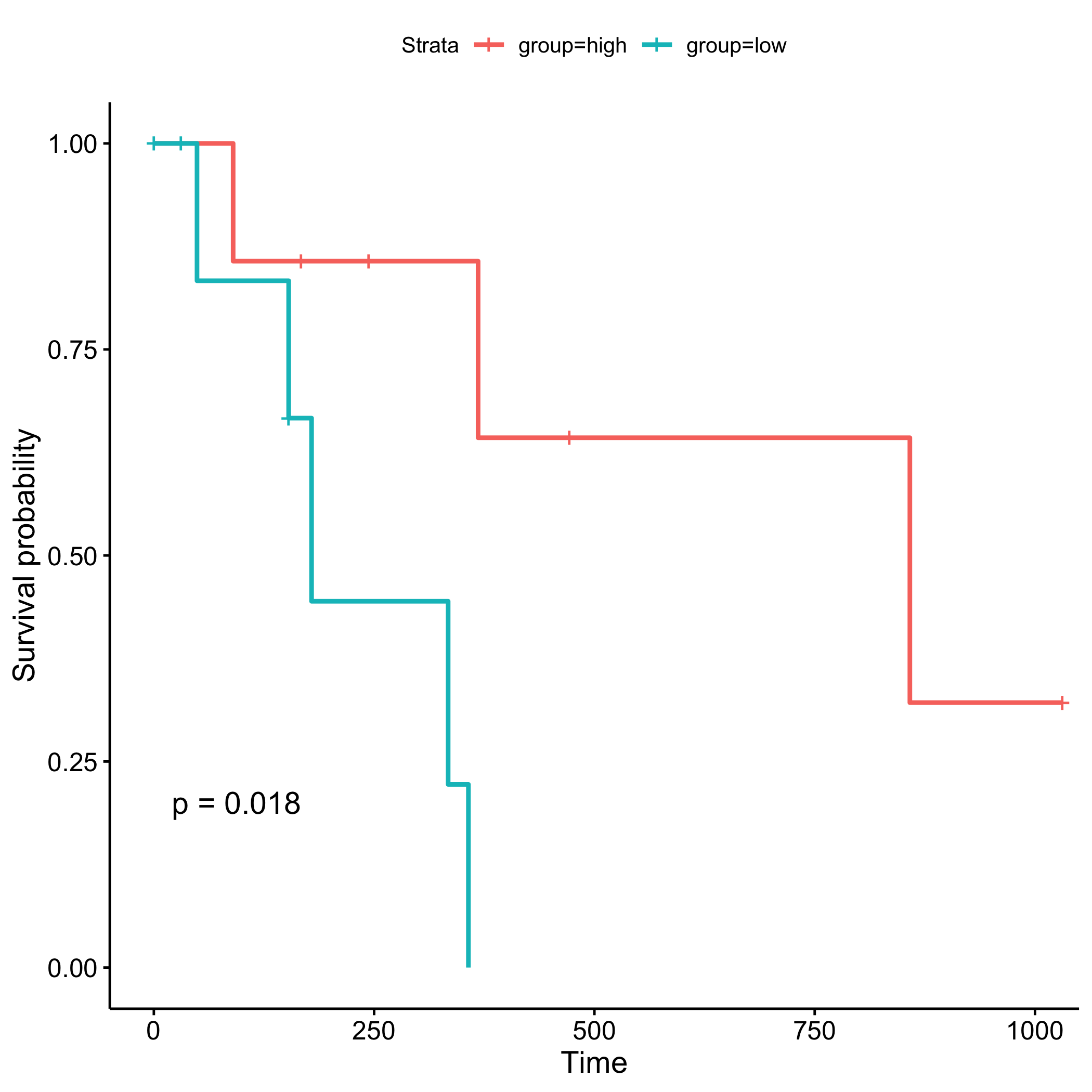

Supplement: Supplementary file 2 — Additional file 2. This data file includes the results of all differentially expressed genes when comparing each tumor location and stage. This file also includes all results from the pathway enrichment analysis that are included in the visualization (Figure 2). Additionally, the survival analysis from all genes with a significant impact on survival is included in this file. [file 12885_2020_6513_MOESM2_ESM.zip › 6.Right.meta.low.C3R4.png]

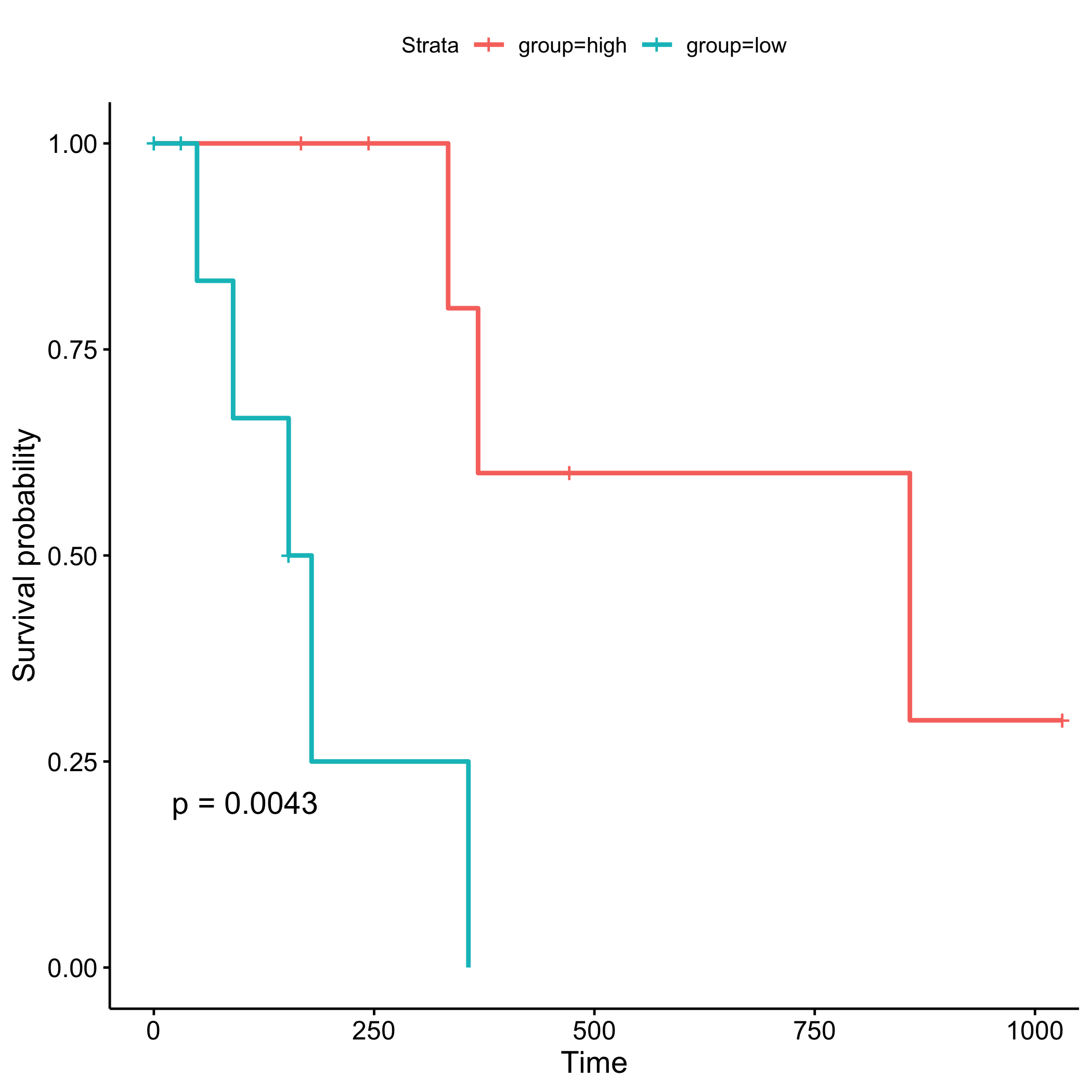

Supplement: Supplementary file 2 — Additional file 2. This data file includes the results of all differentially expressed genes when comparing each tumor location and stage. This file also includes all results from the pathway enrichment analysis that are included in the visualization (Figure 2). Additionally, the survival analysis from all genes with a significant impact on survival is included in this file. [file 12885_2020_6513_MOESM2_ESM.zip › 6.Right.meta.low.CD300AR4.png]

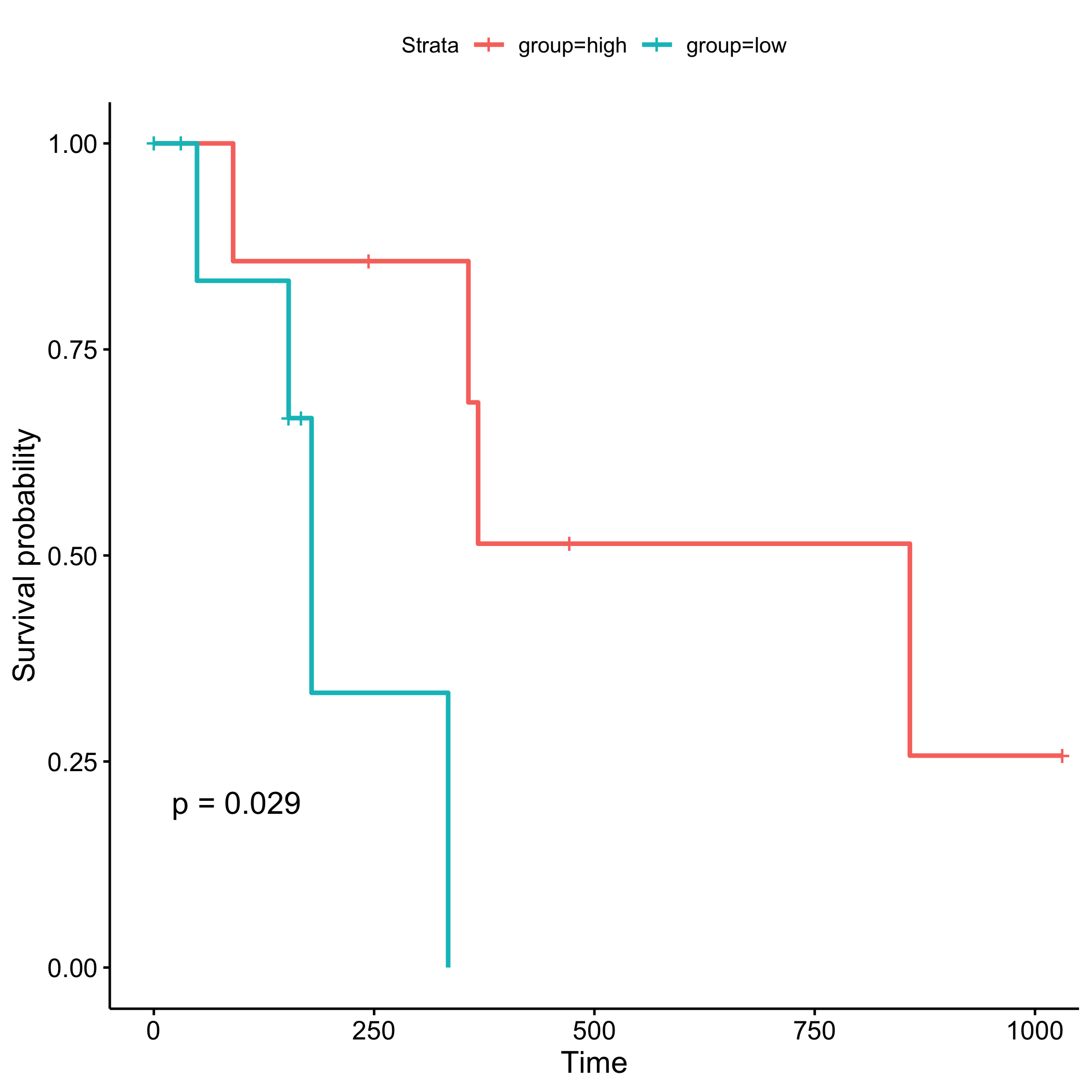

Supplement: Supplementary file 2 — Additional file 2. This data file includes the results of all differentially expressed genes when comparing each tumor location and stage. This file also includes all results from the pathway enrichment analysis that are included in the visualization (Figure 2). Additionally, the survival analysis from all genes with a significant impact on survival is included in this file. [file 12885_2020_6513_MOESM2_ESM.zip › 6.Right.meta.low.CD40LGR4.png]

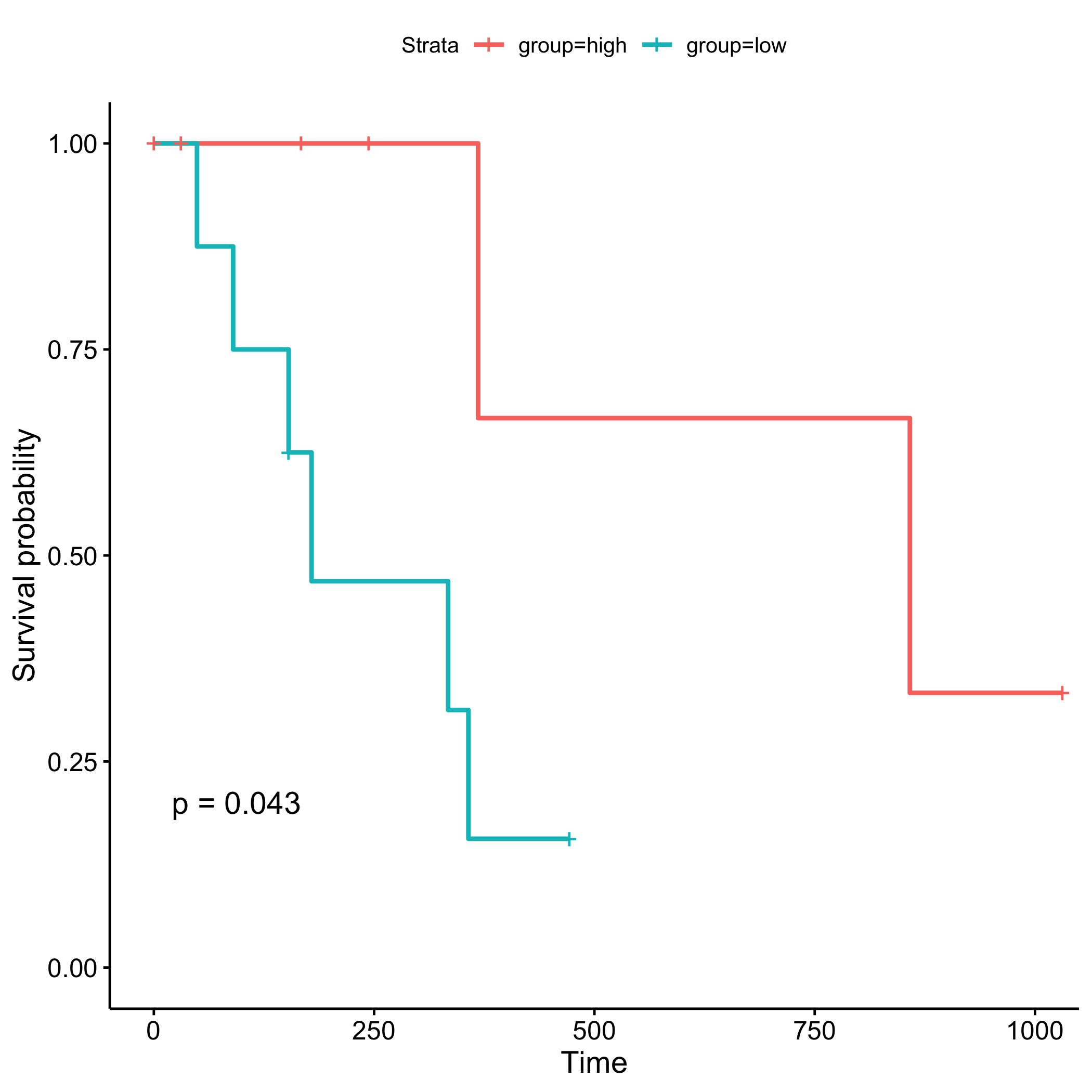

Supplement: Supplementary file 2 — Additional file 2. This data file includes the results of all differentially expressed genes when comparing each tumor location and stage. This file also includes all results from the pathway enrichment analysis that are included in the visualization (Figure 2). Additionally, the survival analysis from all genes with a significant impact on survival is included in this file. [file 12885_2020_6513_MOESM2_ESM.zip › 6.Right.meta.low.CD48R4.png]

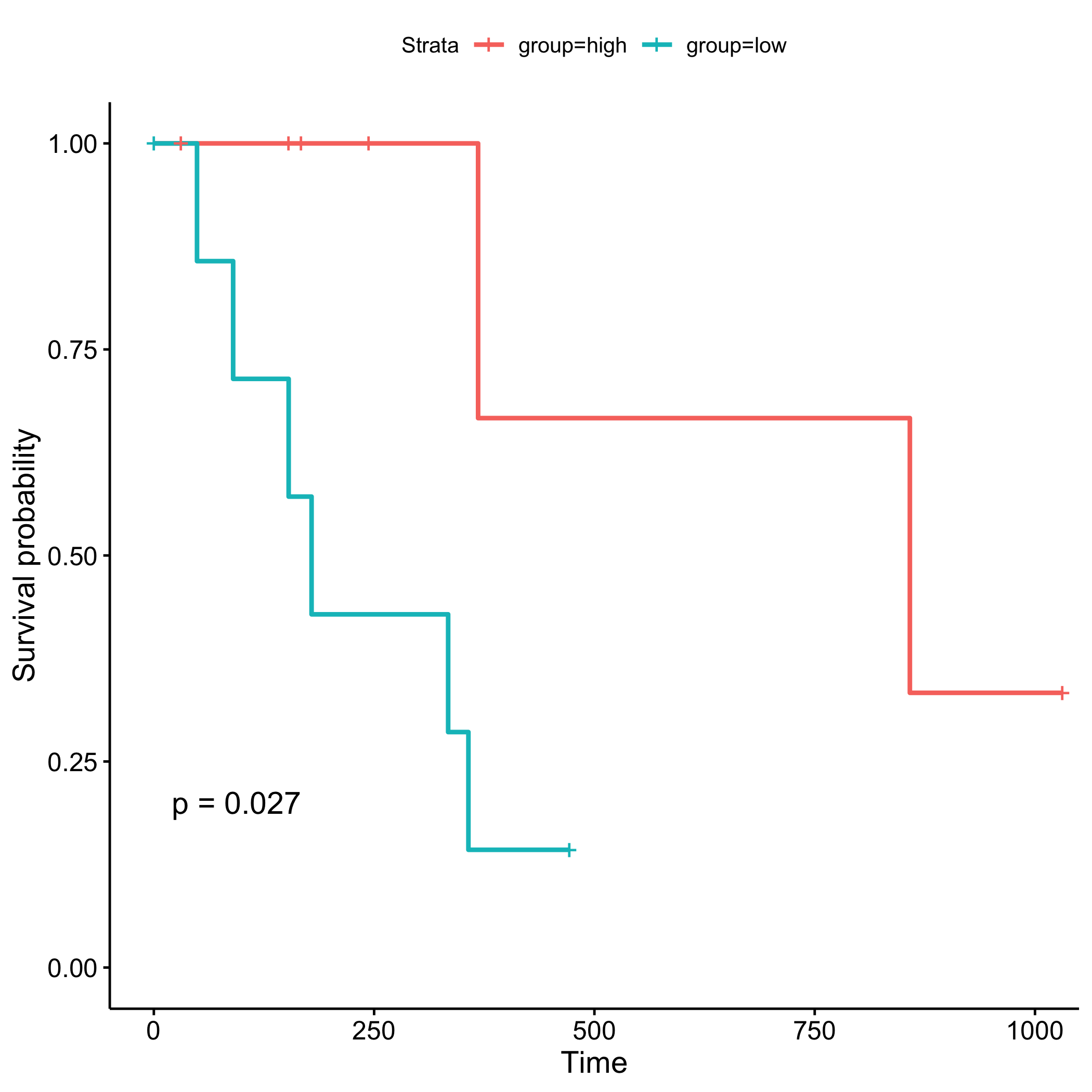

Supplement: Supplementary file 2 — Additional file 2. This data file includes the results of all differentially expressed genes when comparing each tumor location and stage. This file also includes all results from the pathway enrichment analysis that are included in the visualization (Figure 2). Additionally, the survival analysis from all genes with a significant impact on survival is included in this file. [file 12885_2020_6513_MOESM2_ESM.zip › 6.Right.meta.low.CD96R4.png]

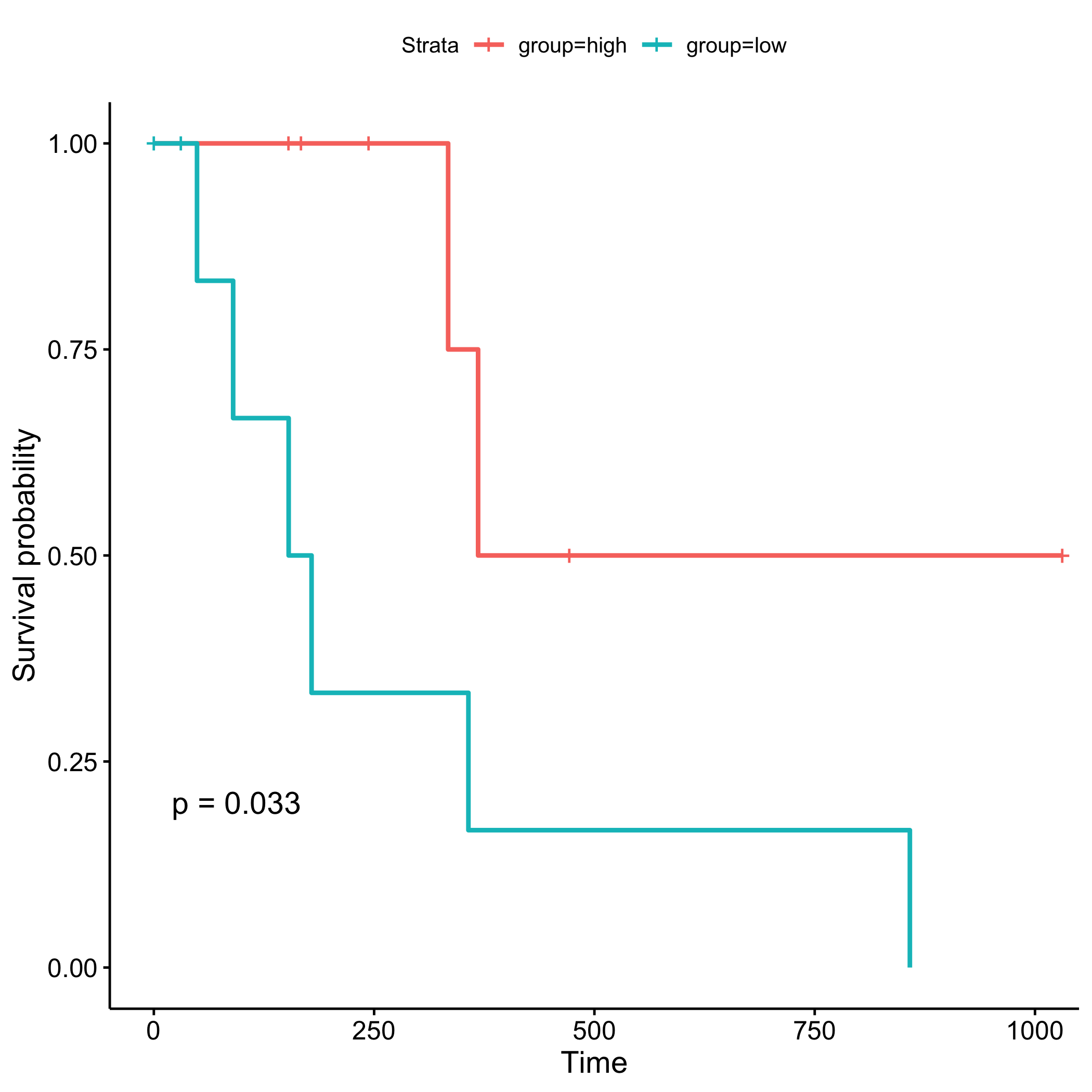

Supplement: Supplementary file 2 — Additional file 2. This data file includes the results of all differentially expressed genes when comparing each tumor location and stage. This file also includes all results from the pathway enrichment analysis that are included in the visualization (Figure 2). Additionally, the survival analysis from all genes with a significant impact on survival is included in this file. [file 12885_2020_6513_MOESM2_ESM.zip › 6.Right.meta.low.ITGB2R4.png]

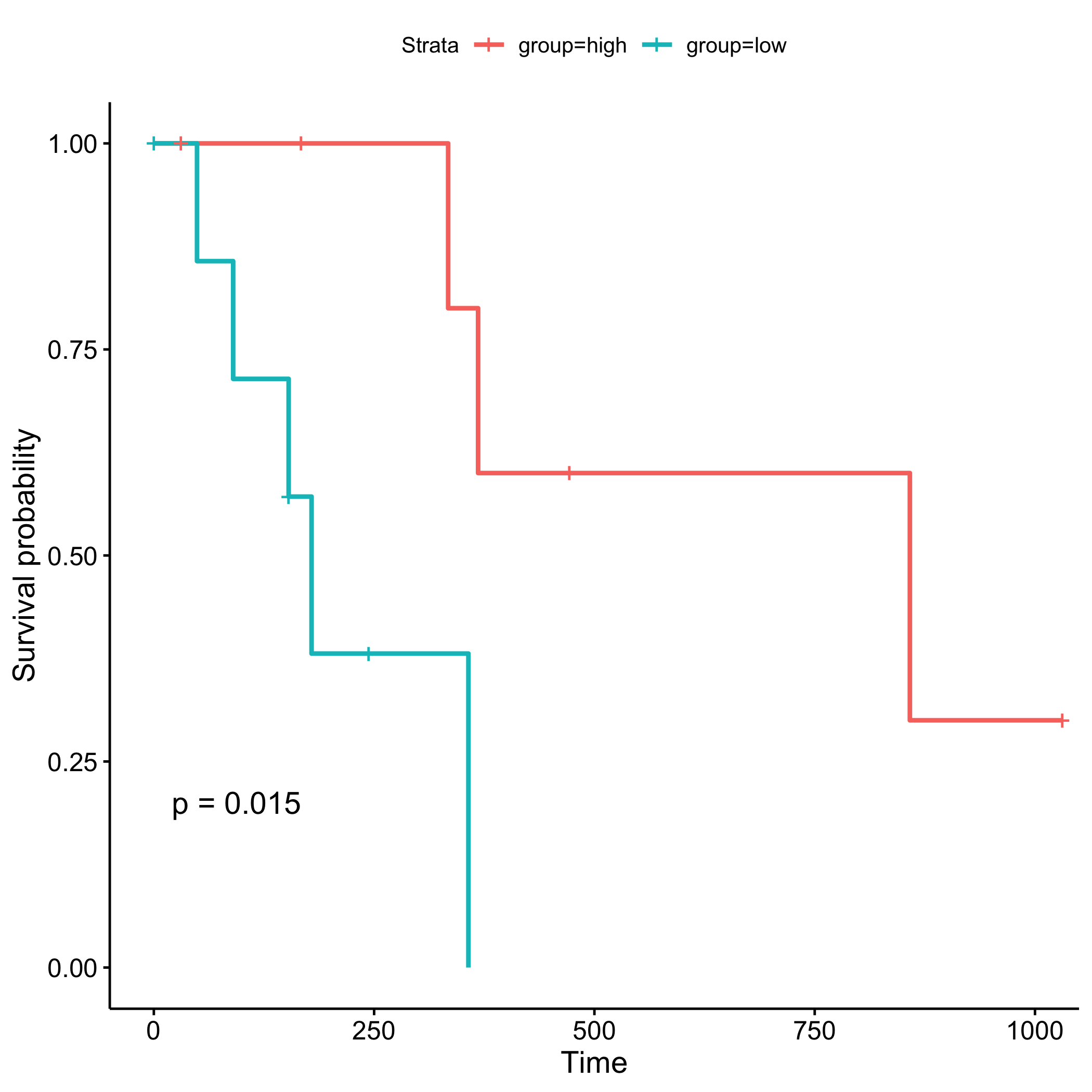

Supplement: Supplementary file 2 — Additional file 2. This data file includes the results of all differentially expressed genes when comparing each tumor location and stage. This file also includes all results from the pathway enrichment analysis that are included in the visualization (Figure 2). Additionally, the survival analysis from all genes with a significant impact on survival is included in this file. [file 12885_2020_6513_MOESM2_ESM.zip › 6.Right.meta.low.LILRB1R4.png]

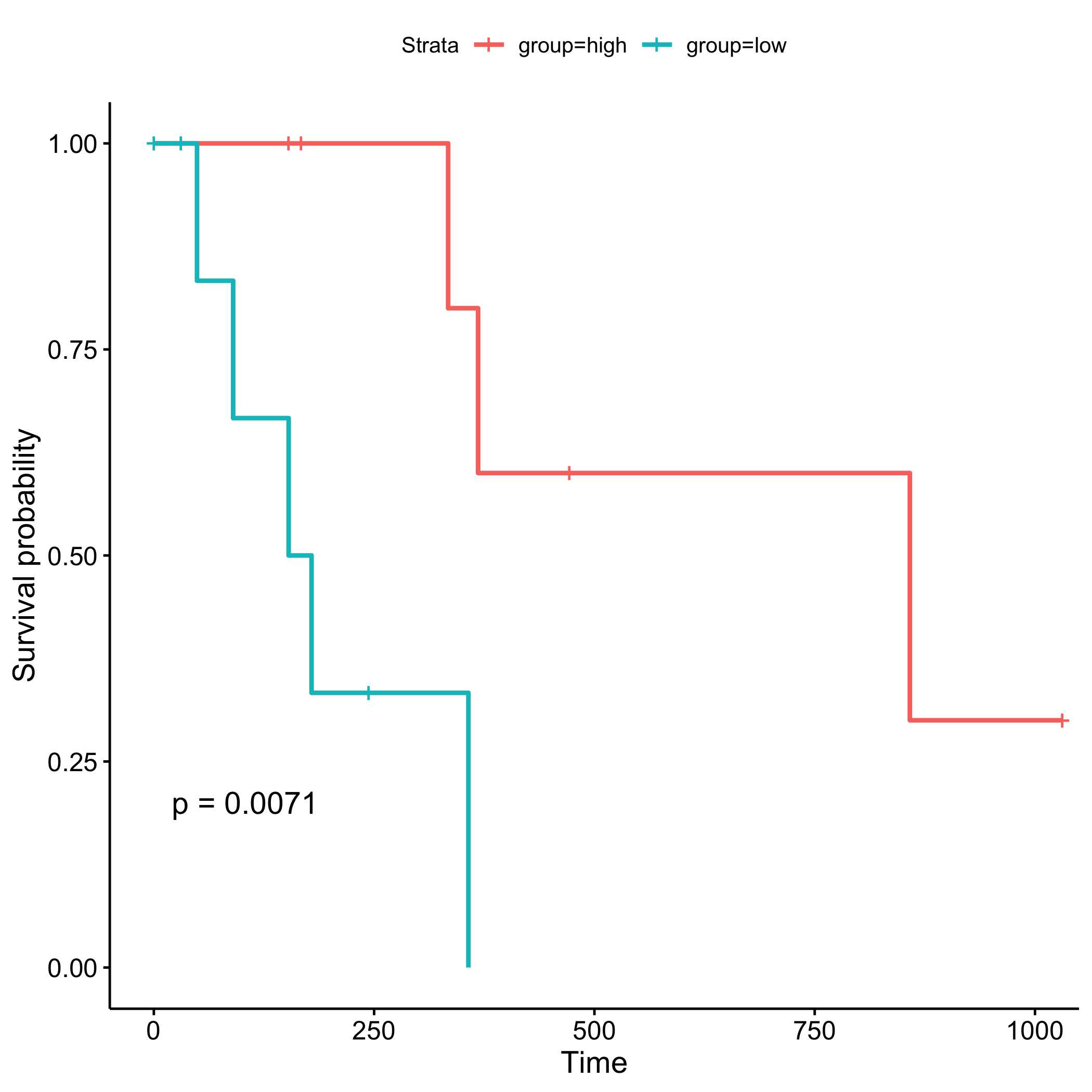

Supplement: Supplementary file 2 — Additional file 2. This data file includes the results of all differentially expressed genes when comparing each tumor location and stage. This file also includes all results from the pathway enrichment analysis that are included in the visualization (Figure 2). Additionally, the survival analysis from all genes with a significant impact on survival is included in this file. [file 12885_2020_6513_MOESM2_ESM.zip › 6.Right.meta.low.SIGLEC9R4.png]
